# A continuum of admixture in the Western Hemisphere revealed by the African Diaspora genome

**DOI:** 10.1038/ncomms12522

**Published:** 2016-10-11

**Authors:** Rasika Ann Mathias, Margaret A. Taub, Christopher R. Gignoux, Wenqing Fu, Shaila Musharoff, Timothy D. O'Connor, Candelaria Vergara, Dara G. Torgerson, Maria Pino-Yanes, Suyash S. Shringarpure, Lili Huang, Nicholas Rafaels, Meher Preethi Boorgula, Henry Richard Johnston, Victor E. Ortega, Albert M. Levin, Wei Song, Raul Torres, Badri Padhukasahasram, Celeste Eng, Delmy-Aracely Mejia-Mejia, Trevor Ferguson, Zhaohui S. Qin, Alan F. Scott, Maria Yazdanbakhsh, James G. Wilson, Javier Marrugo, Leslie A. Lange, Rajesh Kumar, Pedro C. Avila, L. Keoki Williams, Harold Watson, Lorraine B. Ware, Christopher Olopade, Olufunmilayo Olopade, Ricardo Oliveira, Carole Ober, Dan L. Nicolae, Deborah Meyers, Alvaro Mayorga, Jennifer Knight-Madden, Tina Hartert, Nadia N. Hansel, Marilyn G. Foreman, Jean G. Ford, Mezbah U. Faruque, Georgia M. Dunston, Luis Caraballo, Esteban G. Burchard, Eugene Bleecker, Maria Ilma Araujo, Edwin Francisco Herrera-Paz, Kimberly Gietzen, Wendy E. Grus, Michael Bamshad, Carlos D. Bustamante, Eimear E. Kenny, Ryan D. Hernandez, Terri H. Beaty, Ingo Ruczinski, Joshua Akey, CAAPA, Monica Campbell, Sameer Chavan, Cassandra Foster, Li Gao, Edward Horowitz, Romina Ortiz, Joseph Potee, Jingjing Gao, Yijuan Hu, Mark Hansen, Aniket Deshpande, Devin P. Locke, Leslie Grammer, Kwang-YounA Kim, Robert Schleimer, Francisco M. De La Vega, Zachary A. Szpiech, Oluwafemi Oluwole, Ganiyu Arinola, Adolfo Correa, Solomon Musani, Jessica Chong, Deborah Nickerson, Alexander Reiner, Pissamai Maul, Trevor Maul, Beatriz Martinez, Catherine Meza, Gerardo Ayestas, Pamela Landaverde-Torres, Said Omar Leiva Erazo, Rosella Martinez, Luis F. Mayorga, Hector Ramos, Allan Saenz, Gloria Varela, Olga Marina Vasquez, Maureen Samms-Vaughan, Rainford J. Wilks, Akim Adegnika, Ulysse Ateba-Ngoa, Kathleen C. Barnes

**Affiliations:** 1 Department of Medicine, Johns Hopkins University, Baltimore, Maryland 21224, USA; 2 Department of Epidemiology, Bloomberg School of Public Health, JHU, Baltimore, Maryland 21205, USA; 3 Department of Biostatistics, Bloomberg School of Public Health, JHU, Baltimore, Maryland 21205, USA; 4 Department of Genetics, Stanford University School of Medicine, Stanford, California 94305, USA; 5 Department of Genomic Sciences, University of Washington, Seattle, Washington 98195, USA; 6 Institute for Genome Sciences, University of Maryland School of Medicine, Baltimore, Maryland 21201, USA; 7 Program in Personalized and Genomic Medicine, University of Maryland School of Medicine, Baltimore, Maryland 21201, USA; 8 Department of Medicine, University of Maryland School of Medicine, Baltimore, Maryland 21201, USA; 9 Department of Medicine, University of California, San Francisco, San Francisco, California 94143, USA; 10 CIBER de Enfermedades Respiratorias, Instituto de Salud Carlos III, Madrid 28029, Spain; 11 Department of Biostatistics and Bioinformatics, Emory University, Atlanta, Georgia 30322, USA; 12 Center for Human Genomics and Personalized Medicine, Wake Forest School of Medicine, Winston-Salem, North Carolina 27157, USA; 13 Department of Public Health Sciences, Henry Ford Health System, Detroit, Michigan 48202, USA; 14 Biomedical Sciences Graduate Program, University of California, San Francisco, San Francisco, California 94158, USA; 15 Center for Health Policy and Health Services Research, Henry Ford Health System, Detroit, Michigan 48202, USA; 16 Centro de Neumologia y Alergias, San Pedro Sula 21102, Honduras; 17 Faculty of Medicine, Centro Medico de la Familia, San Pedro Sula 21102, Honduras; 18 Tropical Medicine Research Institute, The University of the West Indies, St. Michael BB11115, Barbados; 19 Department of Parasitology, Leiden University Medical Center, Leiden 2333ZA, The Netherlands; 20 Department of Physiology and Biophysics, University of Mississippi Medical Center, Jackson, Mississippi 39216, USA; 21 Instituto de Investigaciones Immunologicas, Universidad de Cartagena, Cartagena 130000, Colombia; 22 Department of Genetics, University of North Carolina, Chapel Hill, North Carolina 27599, USA; 23 Department of Pediatrics, Northwestern University, Chicago, Illinois 60637, USA; 24 The Ann & Robert H. Lurie Children's Hospital of Chicago, Chicago, Illinois 60637, USA; 25 Department of Medicine, Northwestern University, Chicago, Illinois 60637, USA; 26 Department of Internal Medicine, Henry Ford Health System, Detroit, Michigan 48202, USA; 27 Faculty of Medical Sciences Cave Hill Campus, The University of the West Indies, Bridgetown BB11000, Barbados; 28 Queen Elizabeth Hospital, The University of the West Indies, St. Michael BB11115, Barbados; 29 Department of Medicine, Vanderbilt University, Nashville, Tennessee 37232, USA; 30 Department of Pathology, Microbiology and Immunology, Vanderbilt University, Nashville, Tennessee 37232, USA; 31 Department of Medicine and Center for Global Health, University of Chicago, Chicago, Illinois 60637, USA; 32 Department of Medicine, University of Chicago, Chicago, Illinois 60637, USA; 33 Laboratório de Patologia Experimental, Centro de Pesquisas Gonçalo Moniz, Salvador 40296-710, Brazil; 34 Department of Human Genetics, University of Chicago, Chicago, Illinois 60637, USA; 35 Department of Statistics, University of Chicago, Chicago, Illinois 60637, USA; 36 Pulmonary and Critical Care Medicine, Morehouse School of Medicine, Atlanta, Georgia 30310, USA; 37 Department of Medicine, The Brooklyn Hospital Center, Brooklyn, New York 11201, USA; 38 National Human Genome Center, Howard University College of Medicine, Washington DC 20059, USA; 39 Department of Microbiology, Howard University College of Medicine, Washington DC 20059, USA; 40 Institute for Immunological Research, Universidad de Cartagena, Cartagena 130000, Colombia; 41 Department of Bioengineering and Therapeutic Sciences, University of California, San Francisco, San Francisco, California 94158, USA; 42 Immunology Service, Universidade Federal da Bahia, Salvador 401110170, Brazil; 43 Facultad de Medicina, Universidad Catolica de Honduras, San Pedro Sula 21102, Honduras; 44 Illumina, Inc., San Diego, California 92122, USA; 45 Knome Inc., Cambridge, Massachusetts 02141, USA; 46 Department of Pediatrics, University of Washington, Seattle, Washington 98195, USA; 47 Department of Genetics and Genomics, Icahn School of Medicine at Mount Sinai, New York, New York 10029, USA; 48 Institute for Human Genetics, University of California, San Francisco, San Francisco, California 94143, USA; 49 California Institute for Quantitative Biosciences, University of California, San Francisco, California 94143, USA; 50 Data and Statistical Sciences, AbbVie, North Chicago, Illinois 60064, USA; 51 Department of Preventive Medicine, Northwestern University, Chicago, Illinois 60611, USA; 52 Department of Medicine, Northwestern Feinberg School of Medicine, Chicago, Illinois 60611, USA; 53 Department of Chemical Pathology, University of Ibadan, Ibadan 900001, Nigeria; 54 Department of Medicine, University of Mississippi Medical Center, Jackson, Mississippi 39216, USA; 55 Genetics and Epidemiology of Asthma in Barbados, The University of the West Indies, Bridgetown BB11115, Barbados; 56 Faculty of Medicine, Universidad Nacional Autonoma de Honduras en el Valle de Sula, San Pedro Sula 21102, Honduras; 57 Department of Child Health, The University of the West Indies, Kingston 7, Jamaica, Barbados; 58 Centre de Recherches Médicales de Lambaréné, Gabon 13901, Central Africa; 59 Institut für Tropenmedizin, Universität Tübingen, Berlin 72074, Germany

## Abstract

The African Diaspora in the Western Hemisphere represents one of the largest forced migrations in history and had a profound impact on genetic diversity in modern populations. To date, the fine-scale population structure of descendants of the African Diaspora remains largely uncharacterized. Here we present genetic variation from deeply sequenced genomes of 642 individuals from North and South American, Caribbean and West African populations, substantially increasing the lexicon of human genomic variation and suggesting much variation remains to be discovered in African-admixed populations in the Americas. We summarize genetic variation in these populations, quantifying the postcolonial sex-biased European gene flow across multiple regions. Moreover, we refine estimates on the burden of deleterious variants carried across populations and how this varies with African ancestry. Our data are an important resource for empowering disease mapping studies in African-admixed individuals and will facilitate gene discovery for diseases disproportionately affecting individuals of African ancestry.

## Abstract (web summary)

The Consortium on Asthma among African-ancestry Populations in the Americas (CAAPA) aims to better understand population genetics of the African diaspora. Here, it uses deeply sequenced whole-genomes to describe the impact of admixture and potential disease burden of deleterious variants.

## Graphical abstract

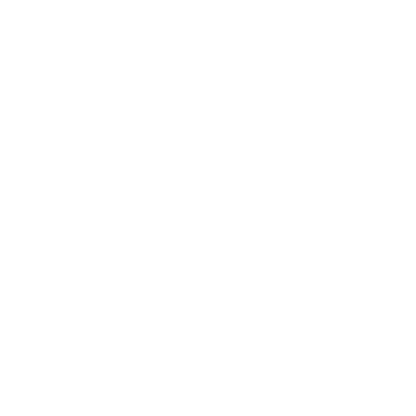

A disproportionate burden of morbidity, disability and death from common, chronic diseases associated with modern lifestyles persists among US racial and ethnic minority populations, most notably among individuals of African ancestry^1^. Unfortunately, the complexity of colonial history has been highly understudied and homogenized: African Americans and admixed populations in Latin America and the Caribbean are grouped into a single racial construct by the American census, which then is often applied in studies of health disparities. This fails to capture the distribution of genetic variation among these populations^2^. In medical genetics, this is especially problematic, where studies of populations of African descent in the Americas do not adequately account for fine-scale population structure resulting from the components of continental ancestry, in particular the lower linkage disequilibrium and decreased genome-wide array coverage in these populations. Although the Americas have been a source of large genome-wide association studies, populations of African descent continue to be understudied.

To address issues of genome-wide genetic diversity, ancestry and admixture, and limitations in commercial genome-wide association studies array coverage in populations of African descent in the Americas (representing the African Diaspora), we performed the largest whole-genome sequencing (WGS) study to date on populations with African ancestry in the Americas. We sequenced 642 unrelated individuals who self-reported African ancestry from 15 North, Central, and South American and Caribbean populations plus Yoruba-speaking individuals from Ibadan, Nigeria, as part of the Consortium on Asthma among African-ancestry Populations in the Americas (CAAPA^3^). These data substantially increase the lexicon of known human genomic variation and suggest an abundance of variation remains to be discovered with more studies of African-admixed populations in the Americas. We summarize genetic variation resulting from the African Diaspora across the Americas and into the Caribbean, quantifying the post-colonial sex-biased European gene flow across multiple regions. Moreover, leveraging our high-coverage whole-genome data we are able to refine estimates on the burden of deleterious variants carried across populations and how this varies with African ancestry. Our data will serve as an important resource for empowering disease mapping studies in African-admixed individuals and facilitate gene discovery for diseases disproportionately affecting individuals of African ancestry.

## Results

### Study design overview

**Figure 1:**
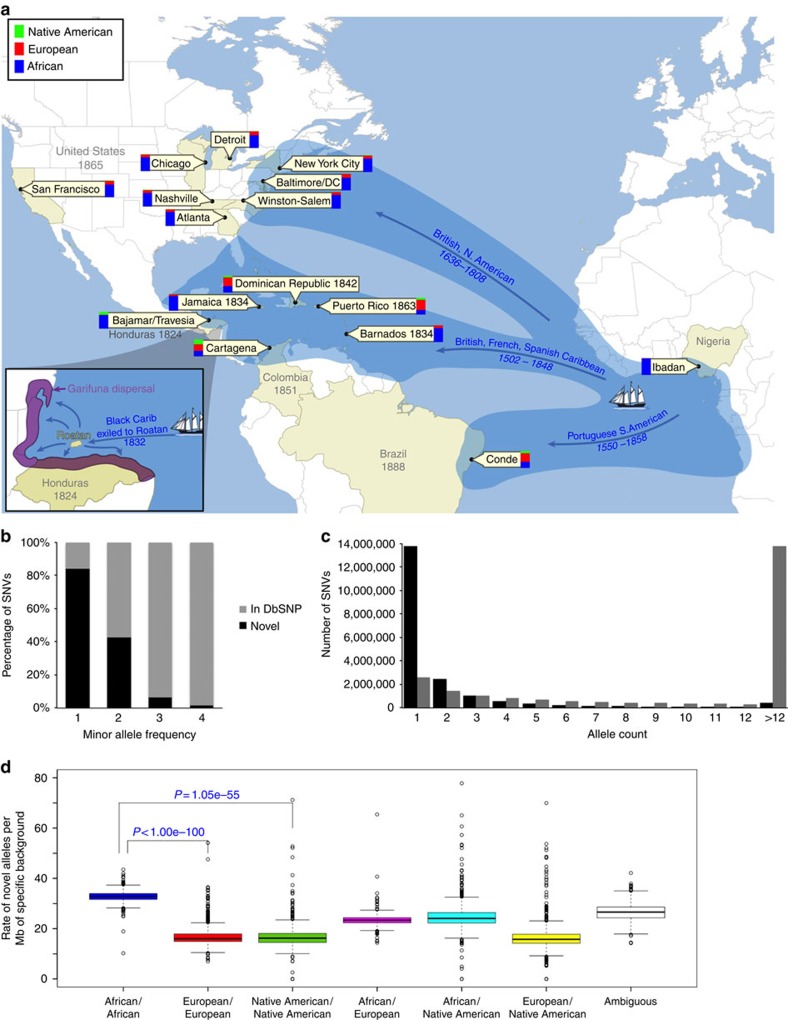
Whole-genome sequences of African-admixed populations in the Americas. (**a**) Geographical location of 16 CAAPA sites and estimates of global ancestry across 642 samples from North, Central and South America and Africa. The transatlantic slave trade is illustrated for each colonial power, along with beginning and end years of the transatlantic slave trade for British/North American, British, French and Spanish Caribbean, and Portuguese/South America. The date of abolition of slavery noted for each country participating in the transatlantic slave trade. The bars depict the relative proportions of African (blue), European (red) and Native American (green) contribution at each CAAPA site. (**b**) Percentage of SNVs within MAF categories (1=singletons, 2=MAF<1%, 3=1%≤MAF≤5%, 4=MAF>5%) across all CAAPA sites illustrating discovery of novel variants (that is, those not previously annotated in dbSNP) across all ranges of MAF. (**c**) Site-frequency spectrum of known and novel SNVs within CAAPA. (**d**) De-convolution of novel alleles by ancestral background in CAAPA using a paired *t*-test illustrates an excess of novel alleles occurs on the African/African background in contrast to the European/European and Native American/Native American background.

The geographic locations of the 15 North, Central, and South American and Caribbean populations sequenced for this study are illustrated in Fig. 1a and detailed information on each of the 15 individual component populations is described in detail in Supplementary Note 1. Supplementary Notes 2–9, Supplementary Figs 1–3 and Supplementary Tables 1 and 2 contain detailed information on sequencing and quality-control pipelines, asthma status, ancestry, observed genetic variation, sequencing depth and call rates by sampling site/ethnicity. Although designed as a case–control study for asthma and associated phenotypes, the systematic characterization of ancestry in all individuals has merit on its own. Asthma is a disease of moderate heritability^4^,^5^, yet few loci have been discovered in populations of African descent (note as exceptions refs 6, 7), and contrasting the study sites yields large sociocultural and environmental heterogeneity that could affect asthma risk. Therefore, across the entire genome, patterns of variation will tend to receive little confounding from our case–control design.

### Characterization of novel variation

Among these deeply sequenced samples, we observed 43.2 million bi-allelic autosomal single-nucleotide variants (SNVs, described in Supplementary Tables 3–7), a greater number than reported from low-coverage sequencing of 1,092 worldwide samples (38 million, from the 1000 Genomes Project (TGP)^8^). A large fraction of these SNVs (*N*=20.7 million) were unique to CAAPA. Of the 43.2 million total SNVs, 16.3 million (38%) were singletons (that is, observed in only a single individual), 12.4 million (29%) were observed in >1 individual but at a minor allele frequency (MAF) <1%, 6.5 million (24%) had 1%≤MAF≤5% and only 7.8 million (29%) of all SNVs were common (MAF>5%), consistent with previous reports^9^,^10^,^11^. Deep sequencing within CAAPA reveals new variants across all categories of MAF (Fig. 1b,c), with a significant excess rate of novel variant discovery on African segments of the genome (Fig. 1d). There were 13.8 million novel singleton variants, 5.3 million novel variants observed in >1 individual but at a MAF <1%, 429,721 had 1%≤MAF≤5% and only 117,367 novel SNVs were common (MAF>5%). Rarefaction curves for various classes of alleles (see Methods and Supplementary Fig. 4) and jackknife projections^12^ suggest if our sample size were doubled, we would discover 68% more apparently damaging coding SNVs (defined by PolyPhen2, see Methods) and 57% more deleterious SNVs genome wide (defined by PhyloP_NH_ score, see Methods). Importantly, with larger sample sizes, we expect to discover deleterious variants at a higher rate than selectively neutral variants, as the former should have lower average MAFs.

### Variation captures population structure and history

**Figure 2:**
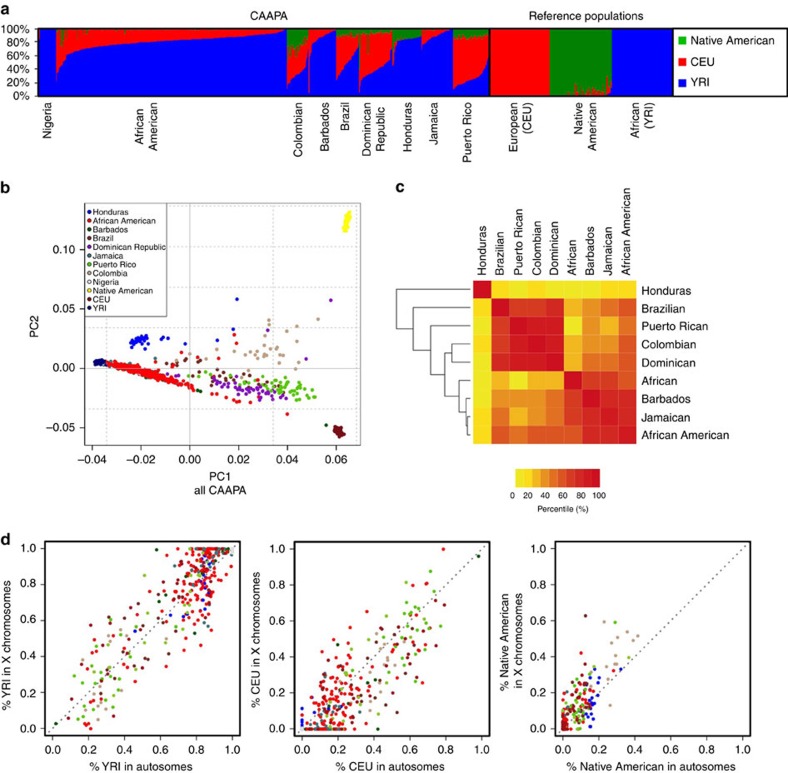
Genomic portraits of admixture heterogeneity within and between populations. (**a**) Estimates of global ancestry of the 642 individuals using ADMIXTURE^47^ analysis on a set of 113,090 LD-pruned SNPs and 3 ancestral reference populations (CEU samples Utah from TGP to represent European ancestry; YRI Yoruban samples from TGP to represent African ancestry; and Native American samples from Mao *et al*.^46^). (**b**) Principal component analysis (EIGENSOFT^48^) using this same set of SNPs and ancestral reference populations illustrating the two main axes of genetic variation in all 642 samples. (**c**) Heat map of doubleton sharing by population; colour is based on the percentiles of the number of doubletons per individual-pair from the same population or from different populations. (**d**) Correlation between autosomal and X chromosome admixture estimates with the identity line in grey (population membership defined as in **b**).

The CAAPA resource represents diverse groups with varying levels of African contributions to ancestry. Relying on three reference ancestral populations^13^ and an optimal *K*=3 (see Methods and Supplementary Fig. 5) global admixture analysis reveals individual autosomal genome-wide estimates of African ancestry ranged from 4% to >99% in CAAPA. The mean African ancestry varied widely among populations from 27% among Puerto Ricans to 89% among Jamaicans in CAAPA groups and approaching 100% as expected among Nigerians (Fig. 2a and see Methods). Principal component analysis (PCA; Fig. 2b and Supplementary Figs 6 and 7) reveals a cluster comprising African American, Barbadian, Jamaican and Nigerian samples along a gradient between European and African ancestral groups, whereas samples from the Dominican Republic, Honduras, Colombia, Puerto Rico and Brazil show more three-way admixture with an average Native American ancestry of 9%, 17%, 28%, 12% and 10%, respectively (Supplementary Table 1). We found minimal differences between all African American populations sampled (Supplementary Fig. 6), consistent with a shared history of many African Americans in the United States^14^. A third component distinguishes the Honduran Garifuna sample from all other CAAPA sites (Supplementary Fig. 7). This component reflects the unique history of the Garifuna (different from other Hondurans described previously^15^), whose ancestors originated from a single slave ship from West Africa that wrecked on the West Indian island of St Vincent in the seventeenth century^16^,^17^,^18^ with subsequent population bottlenecks as described below.

**Figure 3:**
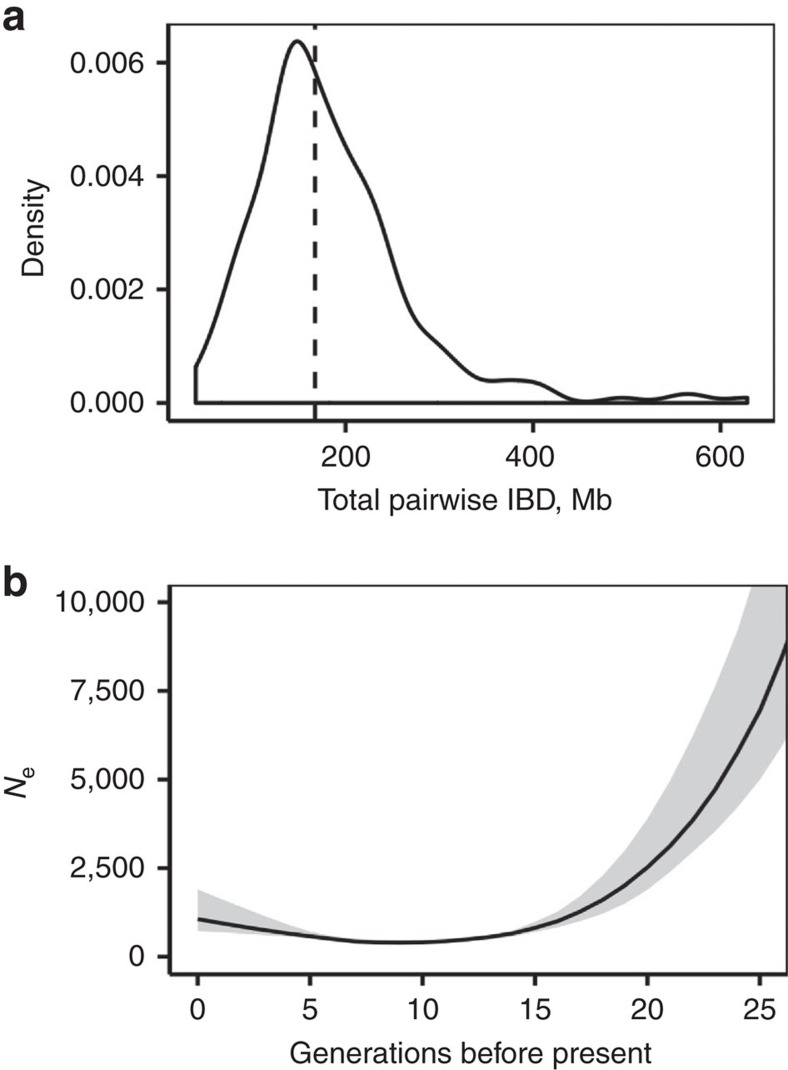
High levels of identity by descent indicate a bottleneck unique to the Honduran Garifuna population. (**a**) Density plot demonstrating elevated pairwise IBD across the Garifuna sample summed across the autosomes. Note: distribution filtered to remove first degree relatives (**b**) Skyline plot of effective population size through time in the Garifuna, as measured from pairwise IBD using the program IBDNe^19^. Line represents maximum likelihood inference, with shaded region the 95% confidence interval determined via bootstrap.

Patterns of rare genetic variation in the CAAPA sequence data recapitulate the complex population history of the Americas. The series of bottlenecks unique to the Honduran Garifuna population^17^,^18^,^19^,^20^ is evidenced by dramatically lower counts of total singletons per individual in this sample (average=15,946 compared with the other sampling sites ranging 26,545–35,565). Consistent with other patterns of bottlenecks in this population, we ran the IBDseq/IBDne pipeline using best practices recommended by the authors^21^. We observe an elevation of median pairwise identity-by-descent (IBD) in the Garifuna (167 Mb), relative to an expected value of 0 Mb for unrelated individuals, as measured using the programme IBDseq across the autosomes (Fig. 3a). Using this distribution of IBD tracts, we could infer recent demographic history (via IBDNe^22^) consistent with a severe bottleneck with recovery beginning 8 generations ago and a minimum effective population size of 395 (95% confidence interval: 352–466; Fig. 3b). Comparing this result with simulations derived from outbred European populations, the observed pairwise IBD values are concordant with the population being as related as second or third cousins. This bottleneck is highly concordant with the historical accounts of the population and helps to characterize the founder effect-driven genetic patterns leading to PC3 in the global data.

Patterns of derived doubleton sharing (capturing shared ancestry at recent mutations) between populations also parallel the proportion of African ancestry and historical records of the slave trade. Specifically, Brazilians, Puerto Ricans, Colombians and Dominicans (with estimated African ancestry ranging from 27 to 49% created by the Spanish–Portuguese slave trade) formed one cluster, and Africans, Barbadians, Jamaicans and African Americans (estimated African ancestry ranging from 76 to 99% created by the British slave trade) formed another, with the unique Honduran Garifuna sharing very little with the other groups (see Methods, Fig. 2c and Supplementary Fig. 8).

### Deleterious variation in coding and non-coding regions

**Figure 4:**
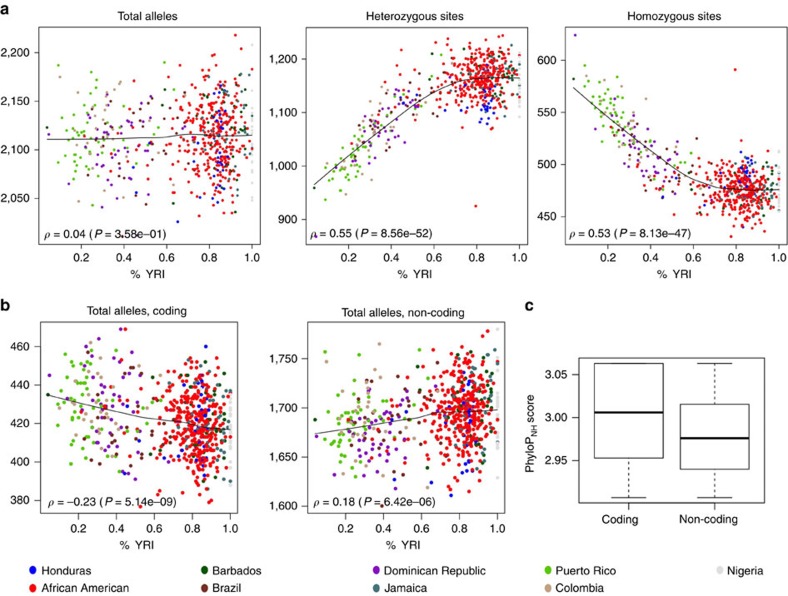
Admixture dynamics influence characteristics of deleterious variation defined by PhyloP_NH_. (**a**) Correlation between the number of total derived alleles, heterozygotes and derived homozygotes of deleterious sites and African ancestry for all samples within CAAPA. (**b**) Correlation between the number of deleterious derived alleles and African ancestry for all samples within CAAPA by coding and non-coding sites. (**c**) Distribution of PhyloP_NH_ scores for coding, and non-coding deleterious sites.

Recent demographic events such as the African Diaspora have clearly affected the frequency spectrum of SNVs in modern populations^8^,^9^,^10^,^11^ but its impact on the average burden of mutations carried by individuals remains more ambiguous. Here we used an unbiased measure of conservation, PhyloP_NH_^23^ to quantify evolutionary constraint and defined deleterious variants as those with PhyloP_NH_ scores exceeding the 99.9th percentile of the empirical distribution of conservation scores across the genome. At this cutoff, 4.2% of all coding variants (including 6.2% of nonsense and 6.6% of non-synonymous variants) and 0.06% of non-coding variants were identified as putatively deleterious. On average, 1,625 deleterious SNVs were carried by each individual, ranging from 1,574 for Puerto Ricans to 1,645 for individuals from Barbados. As expected^24^,^25^,^26^, individuals with more African ancestry carry more predicted deleterious heterozygotes, but those with more European ancestry carry more deleterious derived (compared with the chimpanzee genome) homozygotes, probably a result of the original Out-of-Africa migration (Fig. 4a). These contrasting patterns of deleterious heterozygous and derived homozygous genotypes effectively cancel each other; thus, the average number of deleterious derived alleles per individual is roughly the same with subtle differences as a function of African ancestry (Spearman's correlation between the number of deleterious derived alleles per individual and the proportion of African ancestry was *ρ*=0.04, *P*=0.36; Fig. 4a). These patterns were robust to the metric selected for the definition of ‘deleterious' and similar observations were confirmed when Combined Annotation Dependent Depletion (CADD)^27^ scores were used in conjunction with PhyloP_NH_ (see Methods and Supplementary Fig. 9A). Interestingly, the correlation between the number of deleterious coding alleles per individual and global African ancestry was negative *ρ*=−0.23 (*P*=5 × 10^−9^), whereas it was positive *ρ*=0.18 (*P*=6 × 10^−6^) for deleterious non-coding sites (Fig. 4b). These observations probably reflect differences in the distribution of selective pressure acting on putatively deleterious variants in protein-coding and non-coding regions, as reflected by their PhyloP_NH_ distributions (the median PhyloP_NH_ scores were 3.006 and 2.976 for coding and non-coding deleterious sites, respectively; Mann–Whitney test, *P*=4 × 10^−196^; Fig. 4c). It is important to note that we analysed cases and controls together here: although this could potentially affect patterns of deleterious variants, we assessed sensitivity by restricting the above analysis to controls only (Supplementary Fig. 9B) and observed little difference, both due to small overall genetic differences between cases and controls, and due to the balance of cases and controls across all sub-populations considered here (see Supplementary Table 1).

This opposing correlation pattern remained when we considered the heterogeneity of ancestry contributions across the genome. For example, in each individual, we separately considered sites whose two alleles could be unambiguously inferred to come from the same ancestral population (that is, African and European, respectively, via the local ancestry estimation programme RFMix)^28^. We did not consider sites inferred from Native American ancestry, because 78% of individuals in CAAPA had Native American ancestry estimates of <10%. Next, we compared the proportion of deleterious-derived alleles per individual stratified by local ancestry. Among coding sites, we saw proportionally fewer deleterious alleles in regions of African ancestry compared with European ancestry (that is, 1.33% and 1.41%, respectively; Mann–Whitney test, *P*=0.027). However, in non-coding sites the proportion of deleterious alleles was 0.0294% for African ancestry and 0.0291% for European ancestry (Mann–Whitney test, *P*=6 × 10^−5^; Supplementary Fig. 10), indicating a lower rate of deleterious-derived alleles on segments of African background for coding variants relative to non-coding ones. Thus, these results illustrate how patterns of strongly and weakly deleterious SNVs vary among populations and highlight how both population history and natural selection can influence the burden of deleterious variation and its impact on populations with recently mixed ancestry.

### Evidence for sex-biased gene flow

Historic accounts of mating practices associated with the trans-Atlantic slave trade support sex-biased gene flow in the peopling of the Americas and genetic studies of African-admixed populations in North and South America have shown a significantly higher European male contribution. This process has been documented using genetic data in the past^13^,^26^,^27^,^28^,^29^,^30^. Mating patterns during the African Diaspora varied among colonial regions and we used these CAAPA genomes to characterize differential sex-biased admixture across the 16 CAAPA sites. Comparing estimated admixture fractions of all CAAPA individuals between all autosomes and the X chromosome (see Methods), we see trends similar to previous studies^13^,^26^: female-biased contribution of Native American ancestry (paired *t*-test; *P*=1.2 × 10^−12^), male-biased contribution of European ancestry (paired *t*-test; *P*=8.9 × 10^−12^) and a marginal overall female-biased contribution of African ancestry (paired *t*-test; *P*=0.055; Supplementary Table 8, Fig. 2d and Supplementary Fig. 11). However, these omnibus statistics conflate two separate processes of English and Spanish colonization. African Americans from different US sites exhibited female-biased African and male-biased European trends of admixture (Supplementary Table 8), which agrees with their mitochondrial (maternally transmitted) haplotypes being predominantly African (Supplementary Table 9 and see Methods) and Y-chromosomal haplotypes (paternally transmitted) being predominantly of European origin (Supplementary Table 10 and see Methods). The pattern observed in individuals from Barbados and Jamaica was similar to African Americans, all of whom have a high proportion of African ancestry (Supplementary Fig. 11). The Hondurans' unique history relative to the other Latin American populations is reflected in their higher proportion of African ancestry; in addition, 16% of males carry the only Native American Y-haplotypes seen among CAAPA Latin Americans (Supplementary Fig. 11 and Supplementary Table 10).

Latin American individuals from Brazil, Colombia, the Dominican Republic and Puerto Rico show admixture involving Native American females and European males (Supplementary Table 8 and Supplementary Fig. 11). These patterns of sex-biased ancestry in CAAPA have the same trends as previous studies and some differences may be due to sampling location: Barbadian, Brazilian, Jamaican and Puerto Rican individuals in this study were recruited in their country of origin (Supplementary Table 1) rather than in the United States, which can have its own ancestry-related biases. CAAPA Hondurans and Colombians (from Cartagena, which was one of most active slave ports in Latin America^21^) have a unique history and these specific sub-groups have not previously been included in genetic studies^34^,^35^.

Although the impact of sex-bias in admixed populations of the Americas has been well-established, including among some of the populations included in CAAPA (that is, Brazilian, Colombian and African American), to date to no study has examined sex-bias in as large and diverse a data set as CAAPA (for example, 15 admixed populations across North, Central and South America and the Caribbean). Moreover, there is utility in understanding these processes among populations not recruited in the United States, as has been done in the past, to avoid potential immigration bias. Finally, the ubiquity of these processes across admixed populations within and outside of the United States is noteworthy. It will be of interest to expand these studies, to quantify potentially different admixture processes in Hispanic and non-Hispanic populations.

## Discussion

Leveraging the largest current WGS catalogue of African-admixed individuals from the Americas, we have demonstrated the tremendous genetic variation resulting from the African Diaspora. Despite the large number of novel SNVs carried in individuals of African descent, population history and natural selection have combined to exert subtle impacts on heterozygosity and the burden of deleterious variation. Patterns of genetic distance and sharing of SNVs among these populations reflect the unique population histories in each of the North, Central and South American and Caribbean island destinations of West African slaves, with their particular Western European colonials and Native American populations.

A possible limitation in the study design of CAAPA for examining the pattern of deleterious variants is the selection of subjects on the basis of asthma status (as the long-term goal of this project is to identify genetic determinants associated with risk of asthma among populations of African ancestry). However, very few significant differences were observed between asthmatics and non-asthmatics in global admixture estimates by population (Supplementary Table 1), and a sensitivity analysis restricted to the non-asthmatics revealed no qualitative and only slight quantitative differences in results (Supplementary Fig. 9B).

The complex demographic history present in all the populations in CAAPA can have a significant impact on the genome, particularly in the number of rare variants^10^,^11^,^36^,^37^. How recent events would influence the average burden of apparently deleterious mutations, what proportion of these deleterious mutations actually have true clinical relevance and whether the proportion of deleterious alleles is higher in populations of African ancestry remain unclear. These data underscore the pitfalls of over-homogenizing African ancestry among African-admixed individuals. Identifying a significant excess of novel alleles on chromosomal regions of purely African ancestry (compared with purely European or Native American backgrounds) demonstrates the need for more exhaustive sequencing studies in under-represented racial and ethnic populations to fully catalogue the genetic architecture of disease risk. This, combined with a significant decrease in linkage disequilibrium in African populations^38^, is reflected in the drastically lower coverage of African ancestry variants provided by current commercial arrays of genome-wide markers (see Methods, Supplementary Note 11, and Supplementary Figs 12 and 13). We anticipate the African Diaspora catalogue generated from CAAPA will provide an important and unique reference panel for designing the next generation of genotyping arrays, which will capture a larger percentage of low frequency and rare African variants than currently possible with commercial arrays, providing a more appropriate resource for imputation.

We contend that this WGS data set from 642 individuals of African ancestry representing 16 distinct geographical sites (and peopling histories) is unique and constitutes a novel resource for the scientific community. To this end, the African Diaspora catalogue generated from CAAPA provides an important and unique reference panel for designing the next generation of genotyping arrays, which will capture a larger percentage of low frequency and rare African variants than currently possible with commercial arrays, provide a more appropriate resource for imputation and ultimately facilitate gene discovery for traits in individuals with African ancestry across the world. Major initiatives underway, when combined with CAAPA, will greatly expand the diversity, breadth and power of the African-ancestry genome catalogue (that is, NIH NHLBI TopMed programme^39^, H3Africa Consortium^13^, the Haplotype Reference Consortium^40^) and ultimately facilitate gene discovery for traits in individuals with African ancestry across the world.

## Methods

### Deleterious variant definition

Single-nucleotide polymorphism (SNP) annotation was performed using the SeattleSeq Annotation server^41^; SNPs were annotated as coding-notMod3, coding-synonymous, coding-synonymous-near-splice, intergenic, intron, missense, missense-near-splice, near-gene-3, near-gene-5, splice-3, splice-5, stop-gained, stop-gained-near-splice, stop-loss, utr-3, utr-5 and coding-notMod3-near-splice. We annotated allele ancestry state based on the six primate Endero, Pecan, Ortheus (EPO) alignments and filtered out sites whose ancestral inference had low confidence (that is, ancestral state only supported by one sequence based on the six primate EPO alignments)^8^. Finally, 38,424,038 SNVs were included in the analysis.

Quantification of evolutionary constraints via sequence conservation was widely used to characterize deleterious variants that may have been subject to purifying selection. However, when calculating conservation score when the considering the human reference genome (for example, PhyloP with the human reference genome, PhyloP_H_), a strong bias was observed, as most SNVs where the human genome reference carries the derived allele tend to be classified as ‘benign', regardless of the population frequency^42^,^43^,^44^. To correct this bias, we applied PhyloP_NH_ (PhyloP without the human reference genome) to measure the conservation of genetic sites as previously performed^44^. Briefly, PhyloP_NH_ was based on multiple alignments of EPO 36 eutherian mammal genomes downloaded from Ensembl genome browser and excluding the human reference genome. We defined deleterious variants as those exceeding the 99.9th percentile of PhyloP_NH_ (that is, ≥2.907).

To explore the robustness of our results to our definition of a deleterious variant, we also applied a filter based on CADD score^27^. In this setting, to declare a variant deleterious, we required both PhyloP_NH_≥2.907 and either a CADD cutoff of 30 (corresponding to 99.9th percentile of the genome, in terms of deleteriousness) or a cutoff of 20 (99th percentile of genome).

### PolyPhen2 scores for missense variants

SeattleSeq annotations were used to classify synonymous and non-synonymous SNPs and obtain further functional predictions for each missense variant identified from PolyPhen2 (ref. 45; that is, Probably Damaging, Possibly Damaging and Benign). There have been previous studies documenting strong reference bias existing at sites where the genome reference allele is a derived allele, which results in functional prediction programmes designating a high proportion of these sites as being likely to be non-functional or benign, even when the reference allele is rare in the population overall^42^. To minimize this bias, we filtered out functional designations at sites where the reference allele was derived as unreliable, similar to approaches adopted by Simons *et al*.^42^ To explore robustness of our results to our choice of PolyPhen2, we applied an alternative filter, which combined the PolyPhen2 ‘probably damaging' designation with a SIFT score^46^ cutoff of ≤0.05.

### Rarefaction curves to predict abundance of variation yet to be discovered

As most of the observed variation in CAAPA was novel and rare, we asked whether there are more SNVs to discover as our sample size would increase, or whether the rate of SNP discovery had actually plateaued. Under the standard neutral model of molecular evolution, the number of SNVs discovered is proportional to the partial harmonic series^12^. This function grows logarithmically; thus, it is expected returns would be quite diminished after sequencing ∼500 individuals. In contrast, the non-equilibrium demographic history of modern humans places most populations well off of this curve. We demonstrate this effect using rarefaction curves, which show the fraction of SNVs discovered as a function of sample size across multiple annotations (including the standard neutral model). We then used jackknife projections^12^ to extrapolate the rate of SNV discovery into larger sample sizes, to determine the extent of SNV discovery that would be possible with a larger sample.

### Reference populations used for estimates of admixture

We implemented protocols similar to those established for the TGP reference populations^13^ including the same set of 85 Utah residents with Northern and Western European ancestry (CEU), 88 Yoruba samples from Ibadan, Nigeria (YRI) and 43 Native Americans. The Native Americans were selected from Mao *et al*.^47^ with 99% or higher Native American ancestry estimated by ADMIXTURE^48^. Subsequent to merging the data between CAAPA and these ancestral populations, we obtained a total of 551,510 autosomal SNPs available for analysis; SNPs with >5% missingness were dropped for this final set of merged data. For methods described below that require a set of linkage diseqilibrium-pruned SNPs, we removed SNPs with an *R*^2^-value>0.1 within every 50 SNP window (sliding by 10 SNPs as recommended for ADMIXTURE) and also removed ambiguous SNPs whose strand orientation could not be determined (that is, G/C and A/T SNPs). This yielded a total of 113,090 linkage disequilibrium (LD)-pruned SNPs. Global estimates of admixture were obtained for all 643 independent samples subsequent to the IBD analysis performed above using ADMIXTURE^48^ and including the 3 reference populations. An initial unsupervised analysis was performed with *K*=1–5, to determine the optimal number of ancestral reference groups needed. Setting *K*=3 gave the lowest cross-validation error and this was selected as the *K* under which the final analysis was performed to generate global estimates of ancestry for each sample (Supplementary Fig. 5). We found one African American sample with an estimated African ancestry of 0.001%, that is, essentially no detectable African ancestry, and this sample was dropped from further analysis given its high likelihood of error in DNA plating. The final set of independent samples used in all subsequent analysis was *N*=642.

### Principal component analysis

We used EIGENSOFT^49^ to perform PCA analysis and the R package was used to generate graphical overviews of these results (Supplementary Figs 6 and 7). Primary analysis was performed including all 642 CAAPA subjects and reference populations from 85 CEU, 88 YRI and 43 Native Americans described above. Analysis was also performed on a subset of 328 African Americans and 205 samples from all populations with >5% Native American component within CAAPA, each with the same reference populations. PCA analyses were performed using the set of LD-pruned 113,090 SNPs described above.

### Ancestry estimates by site

We used RFMix^28^ (v1.0.2) to generate local ancestry probabilities from Affymetrix Genome-Wide Human SNP Array 6.0 on CAAPA samples, as well as 85 CEU, 88 YRI and 43 Native Americans from the TGP. The set of 551,510 autosomal SNPs available for analysis in the combined data set were used in the estimation of local ancestry; SNPs with >5% missingness in the combined data set were dropped in this final set of merged data. Data were suitably formatted for BEAGLE^50^, which was used to phase the data for each population in each chromosome. We then used R code to convert BEAGLE output to RFMix format. RFMix was run using Python 2.7 and the Forward–Backward output calculating the posterior probability of each ancestry at each SNP per haplotype. We then used R code to assign ancestral categories from the Forward–Backward output onto the multi-sample vcf file for both alleles at each site per chromosome. Ancestral codes were assigned using the TGP protocol^51^ with 0=unknown, 1=European:European, 2=European:African, 3=African:African, 4=European:Native American, 5=African:Native American and 6=Native American:Native American.

### Doubleton analysis

In total, we observed 3,763,898 derived doubletons with missingness ≤5% for which we observed exactly two copies of derived alleles in 642 individuals. We counted the number of doubletons shared by each individual pair. According to the populations/sampling sites the individual pair belonged to, we normalized the number by the total possible number of individual pairs and summed over all pairs. We generated heat maps using R to exhibit the pattern of doubleton sharing across populations, sampling sites or individuals (Supplementary Fig. 8).

### X-chromosomal admixture analysis

To compare admixture estimates from autosomes and the X chromosome^35^,^52^,^53^, analysis was restricted to only females (to ensure we compared a diploid X to diploid autosomes). We ran ADMIXTURE^48^ at *K*=3 on the X chromosome and autosomes separately as described in Methods (for example, 113,090 LD-pruned SNPs were used for the autosomes and 3,611 LD-pruned SNPs were used for the X chromosome). Mothers transmit an X chromosome to all their children, whereas fathers transmit an X chromosome only to their daughters. Although gender in contemporary individuals has negligible impacts on the long-term evolutionary view, there can be a marked difference in the contribution of ancestral groups. Thus, if females from a specific ancestral population contributed more to current admixed population (that is, female-biased admixture), the admixture fraction estimated from X chromosome SNVs should be larger than seen for SNVs from autosomes for this ancestral population. In contrast, if males from a specific ancestral population contributed more to the admixed population (that is, male-biased admixture), the admixture fraction for SNVs on the X should be smaller than that in autosomes for this ancestral population. To determine sex-biased admixture in the admixed populations, we tested for equality of the ancestral African, European and Native American proportions between the X chromosomes and the autosomes using a paired *t*-test to account for unequal sample variances. As the estimated ancestry proportions are constrained to sum to 1 for each individual for each type of SNV (autosomal or X-chromosomal) and the ancestry estimates for populations are correlated due to their admixture histories, we corrected *P*-values for 57 multiple tests using a conservative Bonferroni correction.

### Mitochondrial haplotypes

To classify mitochondrial haplotypes into haplogroups for the 642 CAAPA males and females, we analysed mitochondrial variant calls with the programme HaploGrep^53^, following phylotree build 16 topology^54^. For sites with low-confidence calls, we manually reviewed haplotypes to confirm haplogroup classifications according to phylotree build 16 (http://www.phylotree.org). Based on mitochondrial DNA phylogeography, individuals from Jamaica, Nigeria and Barbados have almost exclusively sub-Saharan African mitochondrial lineages. Of the 328 African American individuals, 297 (90.5%) have a sub-Saharan African maternal origin based on their mtDNA lineages, 18 (5.5%) have a European origin and 13 (4.0%) have some other origins (Native American, Asian or North African). Of the 205 remaining individuals from Brazil, Colombia, Dominican Republic, Honduras and Puerto Rico, 106 (51.7%) have an sub-Saharan African origin, 80 (39.0%) have Native American lineages and 19 (9.3%) have a European or some other geographical assignations. There are two B4a1a1 and three novel E1a1a sub-lineages present in the sample that might be associated with the Malagasy slave trade and indicates a diverse history of CAAPA individuals^56^. The full list of haplotypes is in Supplementary Data 1.

### Y-chromosomal haplotypes

Y chromosomal haplotypes of CAAPA males in Supplementary Table 10 were determined using a pipeline from Poznik *et al*.^57^ and Haplogrep^54^, and have the following geographical distribution. The B1, B2, E1a and E2 haplotypes are African; E1b haplotypes are most likely to be of African origin given the sampling locations; G, I and J haplotypes are most likely to be European; Q1a is Native American; R1a is Asian; and of the 76 R1b haplotypes, 74 are European and 2 are African^58^. It is notable that three of the four observed Q1a Native American haplotypes are in Hondurans. Nigerians carry exclusively African haplotypes. In African Americans, 59.8% of the haplotypes are African E1b, 20.6% are European R1b and 19.6% are other types that are mostly European. The remaining groups have the following composition of non-European haplotypes. Honduras: 78.9% African, 15.7% Native American; Barbados: 81.8% African; Brazil: 20% African; Colombia: 12.5% African; Dominican Republic: 36.8% African; Jamaica: 60.8% African; and Puerto Rico: 12.5% African.

### Data availability

The WGS data that support the findings of this study have been deposited in dbGAP with the accession code phs001123.v1.p1. All relevant data can be accessed through dbGAP. Specific data use limitations: GRU-IRB (General Research Use, IRB approval required).

## Additional information

**How to cite this article:** Mathias, R. A. *et al*. A continuum of admixture in the Western Hemisphere revealed by the African Diaspora genome. *Nat. Commun.* 7:12522 doi: 10.1038/ncomms12522 (2016).

## Supplementary Material

Supplementary Information Supplementary Figures 1-13, Supplementary Table 1-10, Supplementary Note 1-11 and Supplementary References.
