# Supplementary material for: A continuum of admixture in the Western Hemisphere revealed by the African Diaspora genome
Source: Nat Commun. 2016 Oct 11;7:12522. doi: 10.1038/ncomms12522 (PMC5062574; doi:10.1038/ncomms12522)
Supplement: Supplementary Information — Supplementary Figures 1-13, Supplementary Table 1-10, Supplementary Note 1-11 and Supplementary References. [file ncomms12522-s1.pdf]

Supplementary Figures

Supplementary Fig 1: Estimates of kinship in CAAPA using a set of 490,179 LD-pruned SNPs genotyped on the Illumina Omni 2.5 array on all samples with WGS data showing [A] relationships across pairs derived from all the CAAPA samples (N=661) with sequence data; [B] relationships across pairs derived from N=643 samples after 18 related subjects were excluded; and [C] the within Honduras-pairs relative to the full distribution across CAAPA illustrating the lack of excess endogamy within the Honduras sample. **Note:** An additional sample was dropped based on ancestry for a final N=642 subjects used in all subsequent analysis.

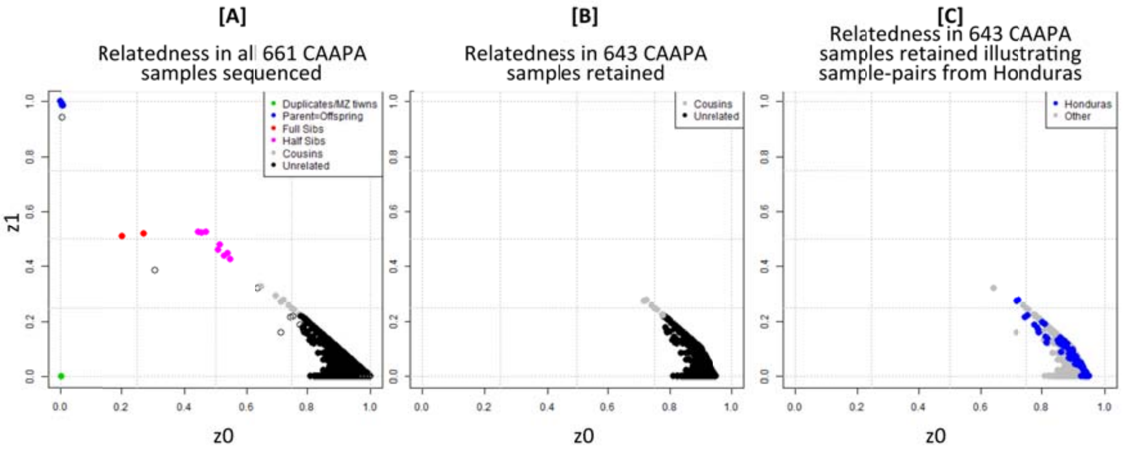

Supplementary Fig 2: Mean detection rate and accuracy for sequencing variant calls across samples for heterozygous (left column) and homozygous alternate (right column) calls on Illumina Omni 2.5 array data for the same individuals, as sequencing quality filters are varied. Shown are comparisons when sequence variants are filtered when below two levels of genotype quality (Q, cutoffs of 20 and 30), or read depth (DP, depth of 7 and 10), and the combination of Q of 20 and the two values of DP.

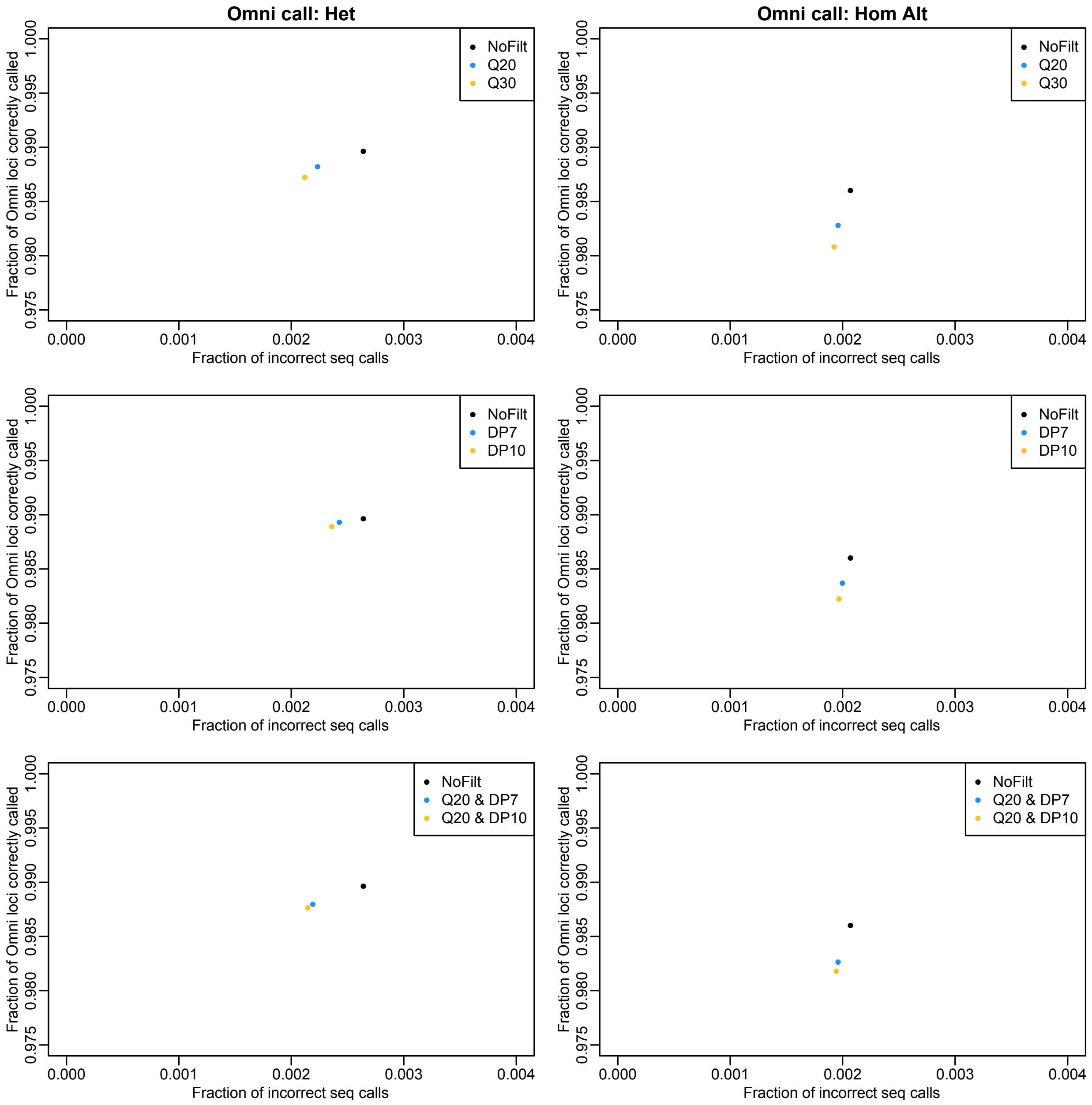
Omni call: Het
Omni call: Hom Alt
Fraction of Omni loci correctly called
0.975
0.980
0.985
0.990
0.995
1.000
Fraction of incorrect seq calls
0.000
0.001
0.002
0.003
0.004
NoFilt
Q20
Q30
DP7
DP10
Q20 & DP7
Q20 & DP10

Supplementary Fig 3: Illustration of variant calling issues within segmental duplication regions. Plot on top left shows the count of samples with a heterozygous call per variant site along chromosome 22 and plot on top right highlights in red all SNPs within a segmental duplication region on chromosome 22. Plot on bottom left shows the number of variant sites called within a sliding window of 50kb prior to filtering segmental duplication regions, and plot on bottom right shows the number of variants sites called within a sliding window of 50kb after segmental duplications were filtered on chromosome 22.

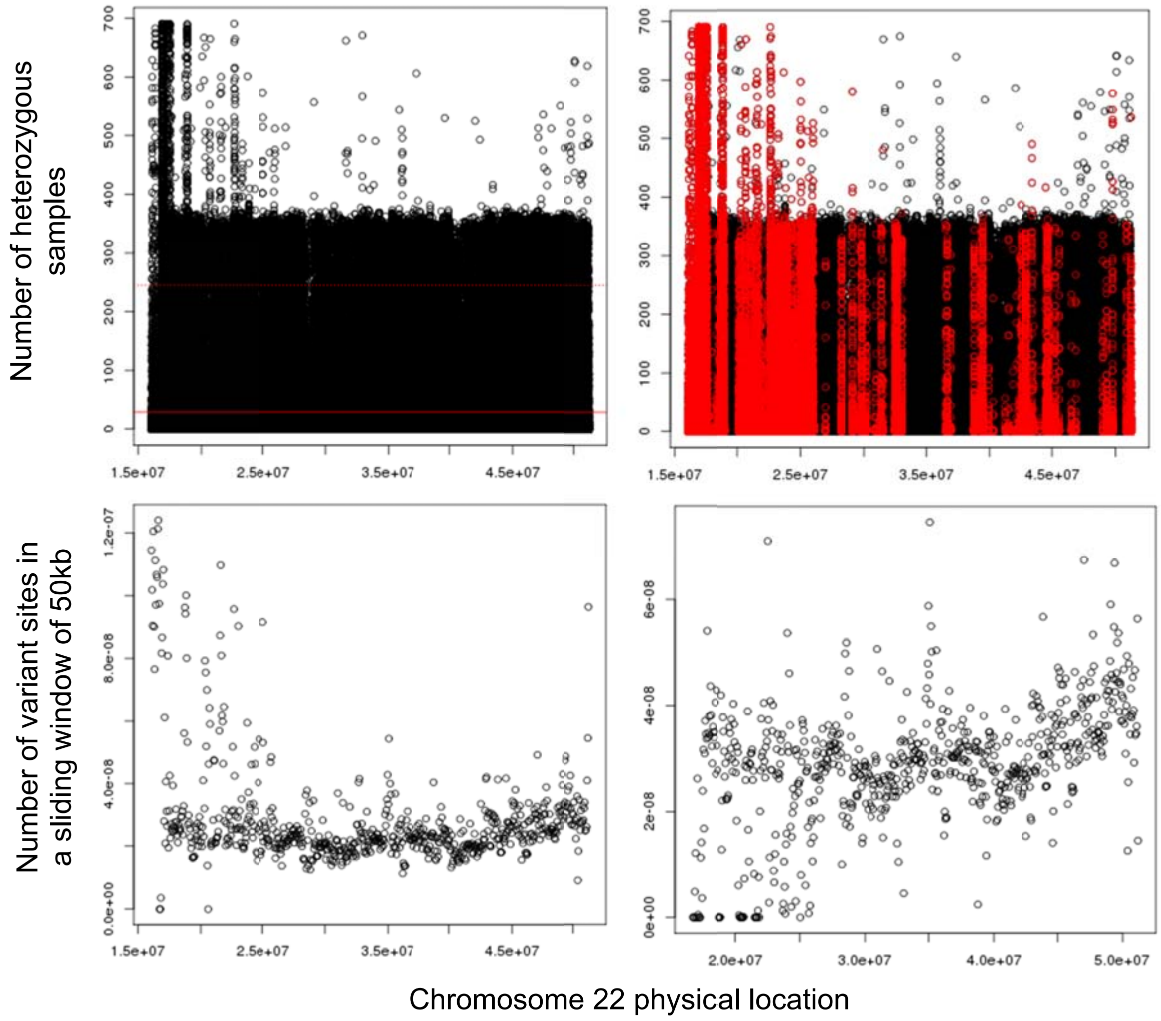

Supplementary Fig 4: Rarefaction analysis of SNV discovery, and jackknife projections into larger sample sizes. The lower left quadrant (light yellow shading) shows the fraction of SNVs discovered as a function of the number of individuals included in our study. The upper right quadrant shows the jackknife extrapolation of our SNV discovery sample into larger sample sizes. In the legend, CDS refers to coding sequence annotation by PolyPhen2.

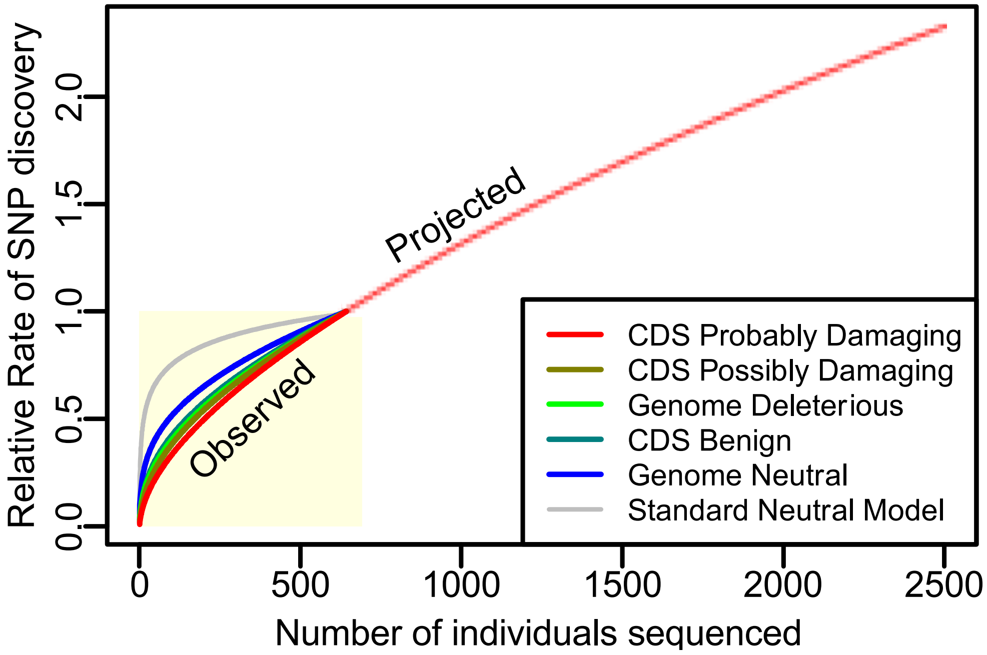

Supplementary Fig 5: Unsupervised ADMIXTURE analysis to determine optimal K for all CAAPA subjects. The most appropriate K was 3.

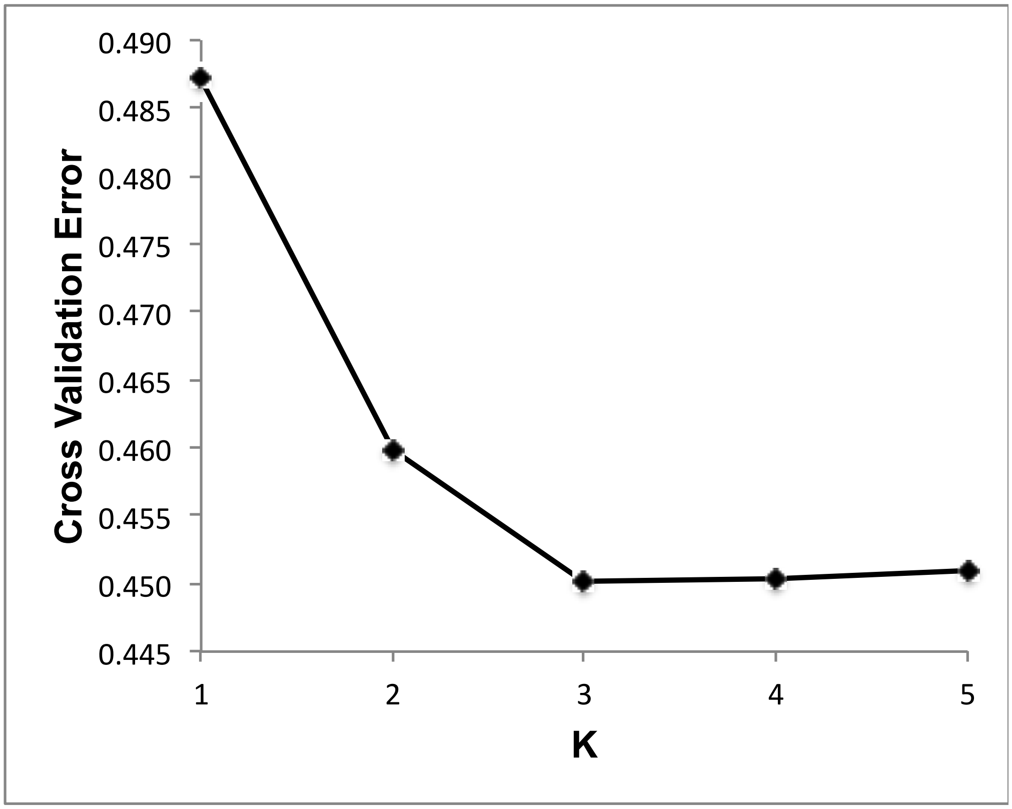

Supplementary Fig 6: Principal components generated by EIGENSOFT[1] using a set of 113,090 LD-pruned SNPs with three ancestral reference populations (CEU samples Utah from TGP to represent European ancestry, YRI Yoruban samples from TGP to represent African ancestry, and Native American samples [2]); illustrating minimal differences between the all African American samples from eight geographic sites across the United States.

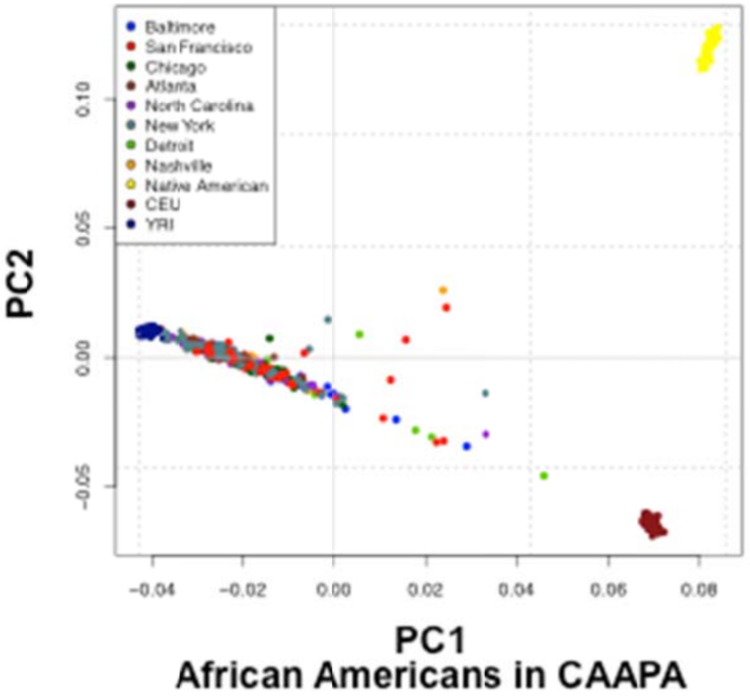

Supplementary Fig 7: Principal component analysis showing PC3 for the Garifuna from Honduras (blue). Panel A shows all 642 CAAPA samples, and Panel B is limited to 205 samples from populations with an average of >5% Native American ancestry. Analysis was performed using a set of 113,090 LD-pruned SNPs and three publicly available ancestral populations (Native Americans[2,3], Utah residents with Northern and Western European ancestry[4] from the U.S. and Yoruba samples from Ibadan, Nigeria[4]) in EIGENSOFT[1] .

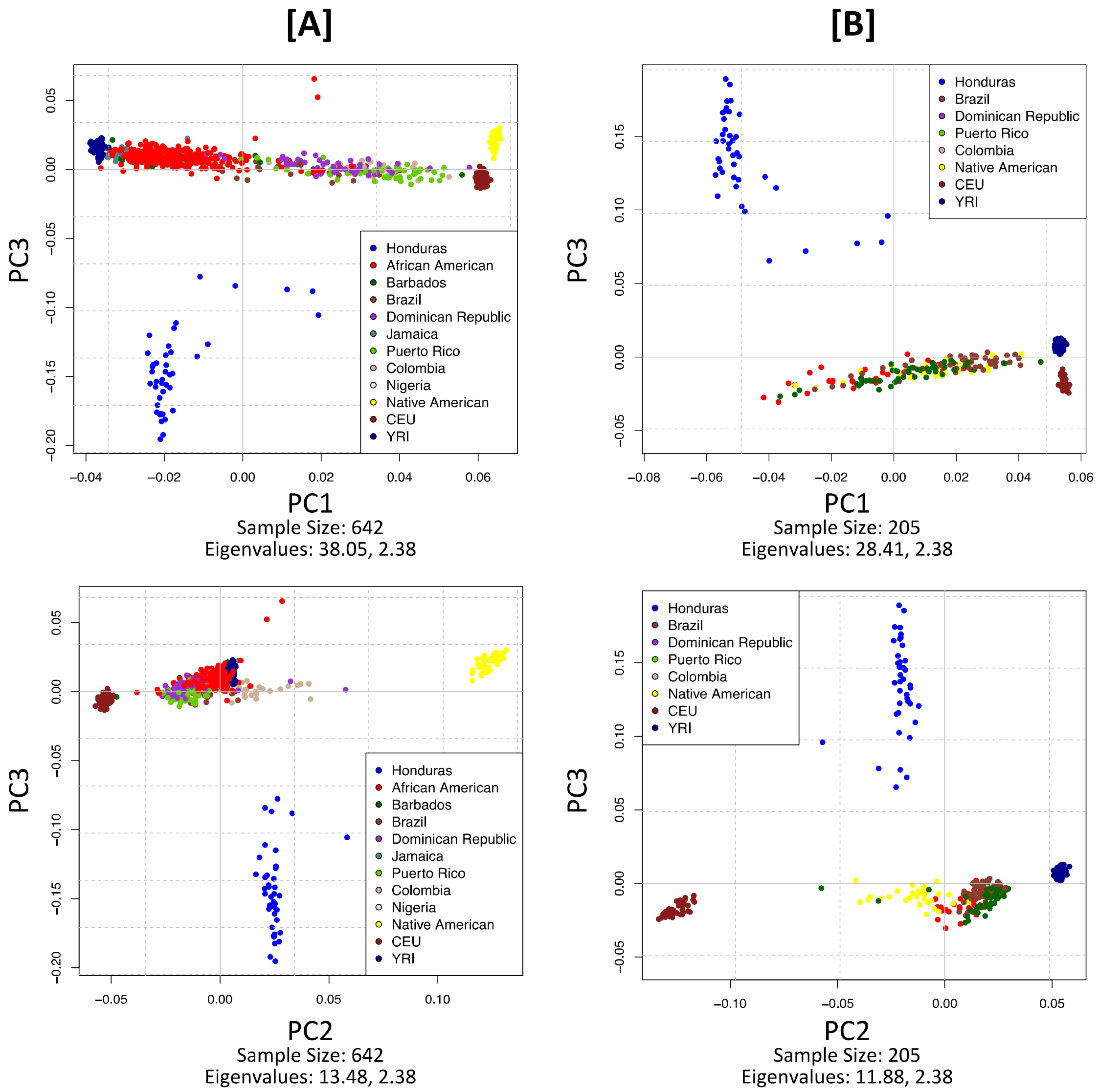

Supplementary Fig 8: Heat map of doubleton sharing. Panel A summarized the number of doubletons per individual pair from the same population or from different populations; Panel B summarized the number of doubletons per individual pair for all African Americans sampled from same site or from different sites. Panel C summarized the number of doubletons shared by each individual pair from all 16 geographic sampling sites. Color is based on the percentiles of the number of doubletons (per individual pair) for each panel, respectively.

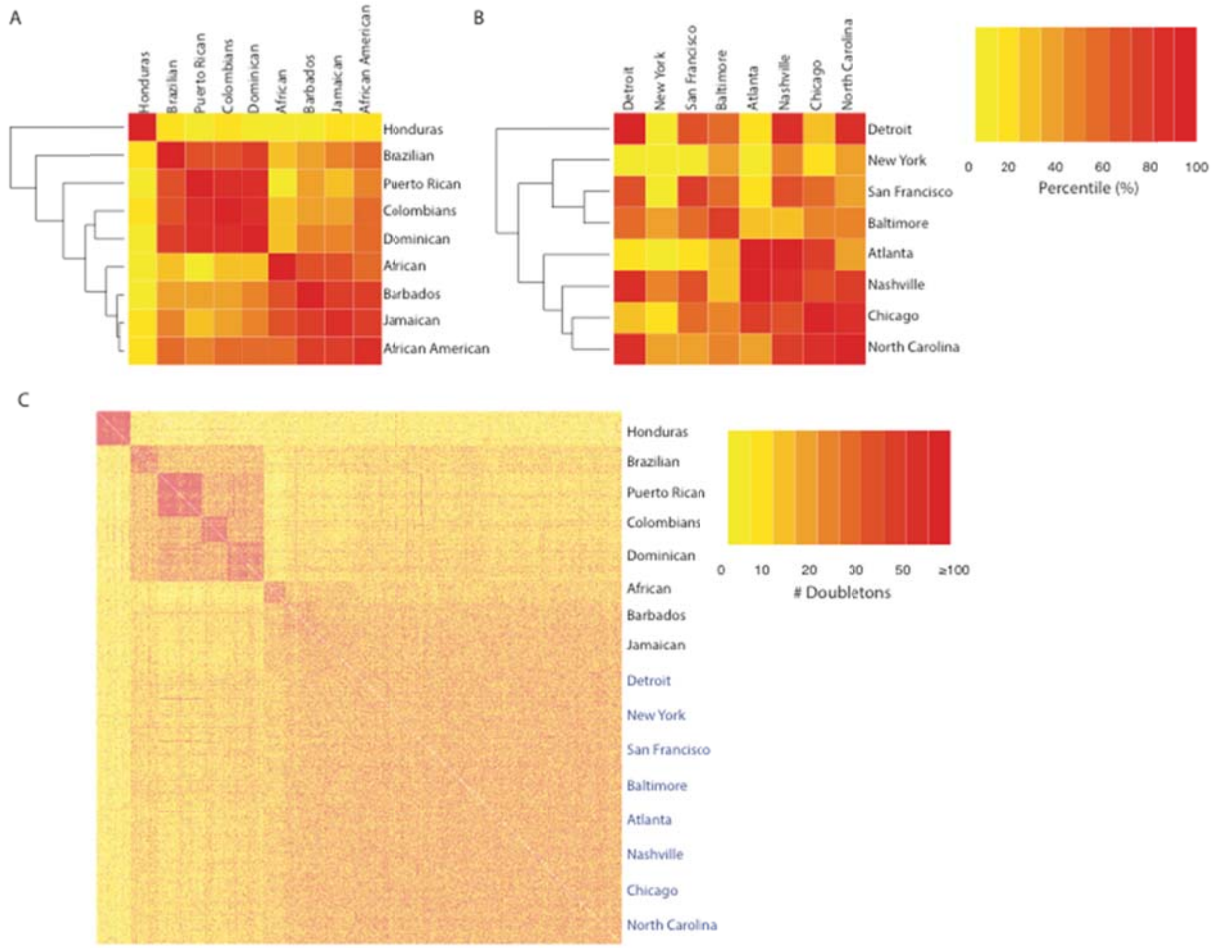

Supplementary Fig 9. Admixture dynamics influence characteristics of deleterious variation. **A.** Deleterious variants defined as a $PhyloP_{NH} \geq 2.907$ and $CADD \geq 20$. Correlation between the number of total derived alleles, heterozygotes and derived homozygotes of deleterious sites and African ancestry for all samples within CAAPA. **B.** Deleterious variants defined as a $PhyloP_{NH} \geq 2.907$, subjects restricted to control samples only. Correlation between the number of total derived alleles, heterozygotes and derived homozygotes of deleterious sites and African ancestry for only control samples within CAAPA.

A.

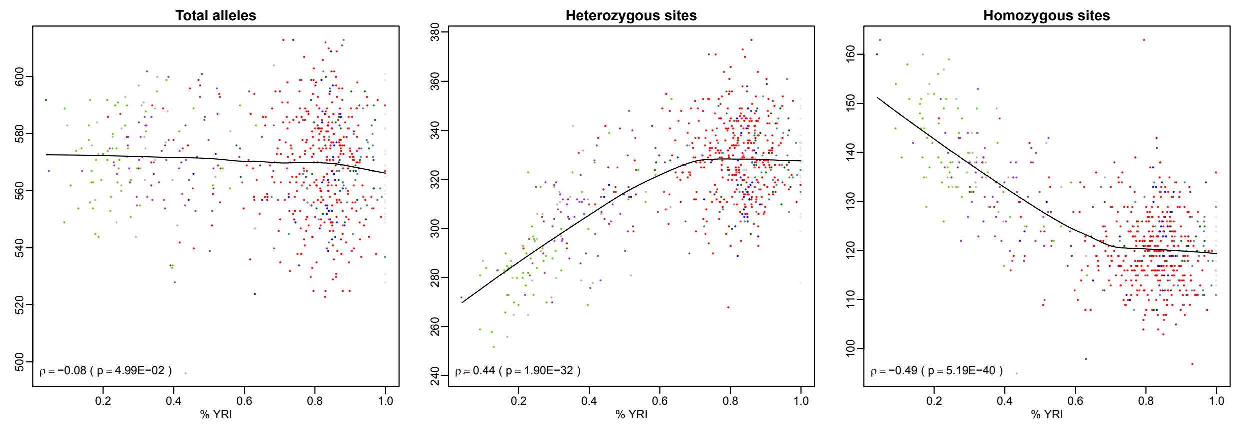

B.

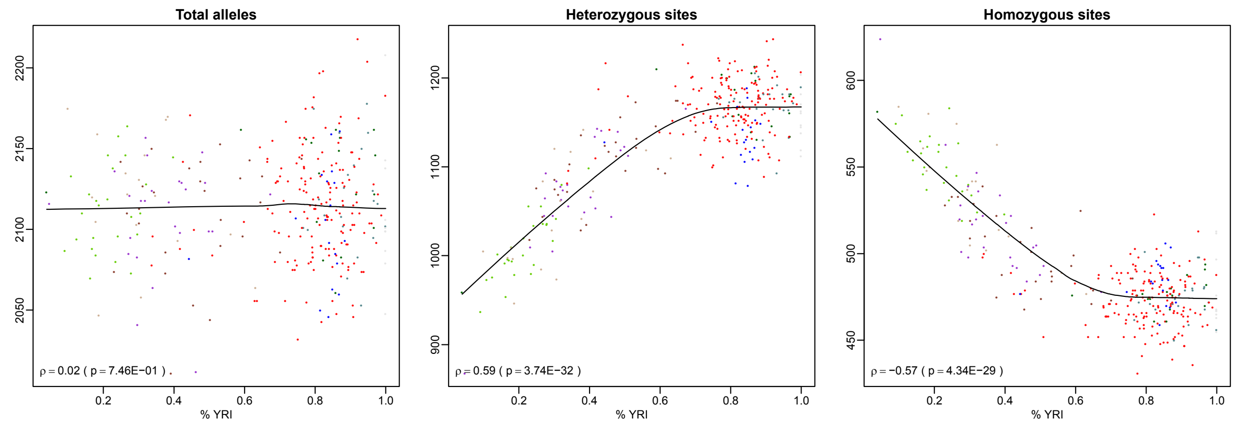

Supplementary Fig 10: The proportion of deleterious derived alleles per individual by ancestral background where both alleles at the SNV were inferred from African ancestry or were inferred from European ancestry. Sites were labeled as 'deleterious' if they fell above the 99.9th percentile of PhyloP$_{NH}$ and further classified as 'coding' and 'noncoding' sites. The proportions represent counts of deleterious derived alleles over the total count of derived alleles in the individual for sites from the same ancestral background.

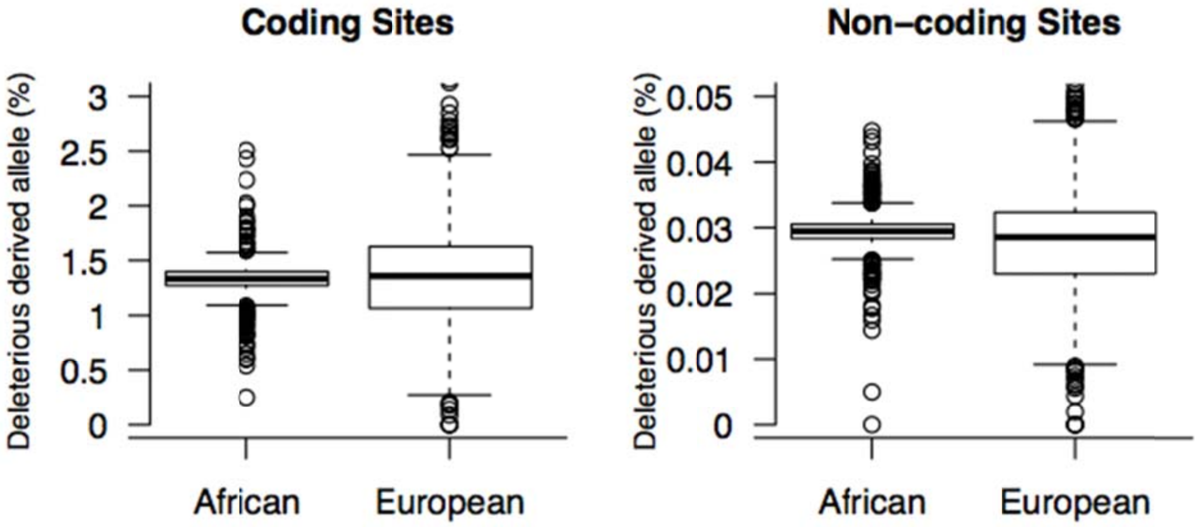

Supplementary Fig 11: ADMIXTURE estimates from autosomes and X chromosome by population within CAAPA. Boxplots are shown separately for X chromosomes and autosomes denoting European (red), African (blue), and Native American (green) ancestry estimates.

(a) African-Americans

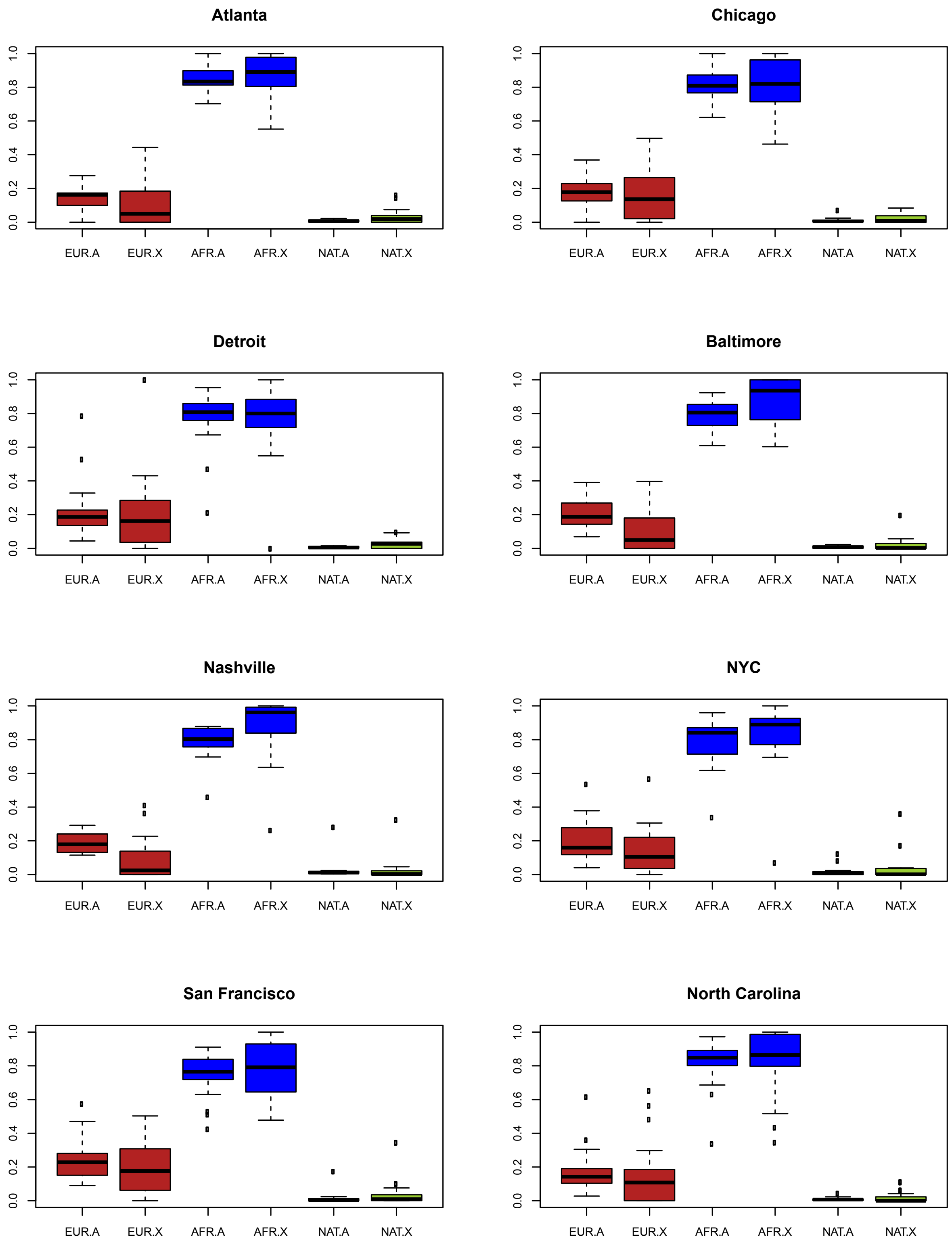

(b) Afro-Caribbean

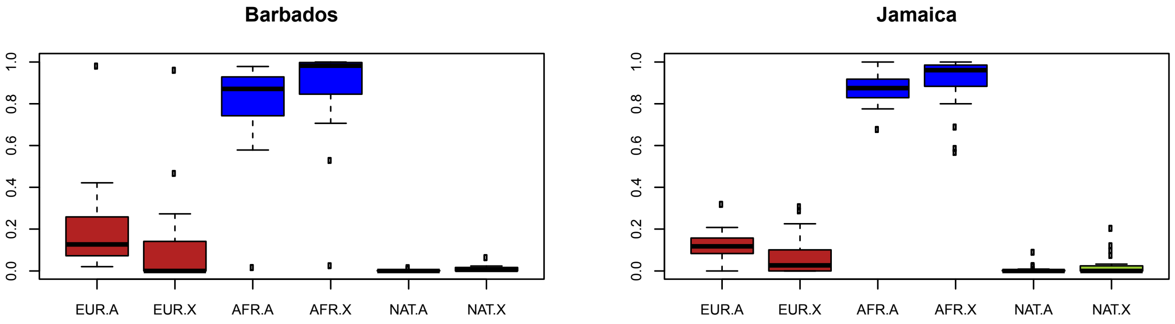

(c) Latin American

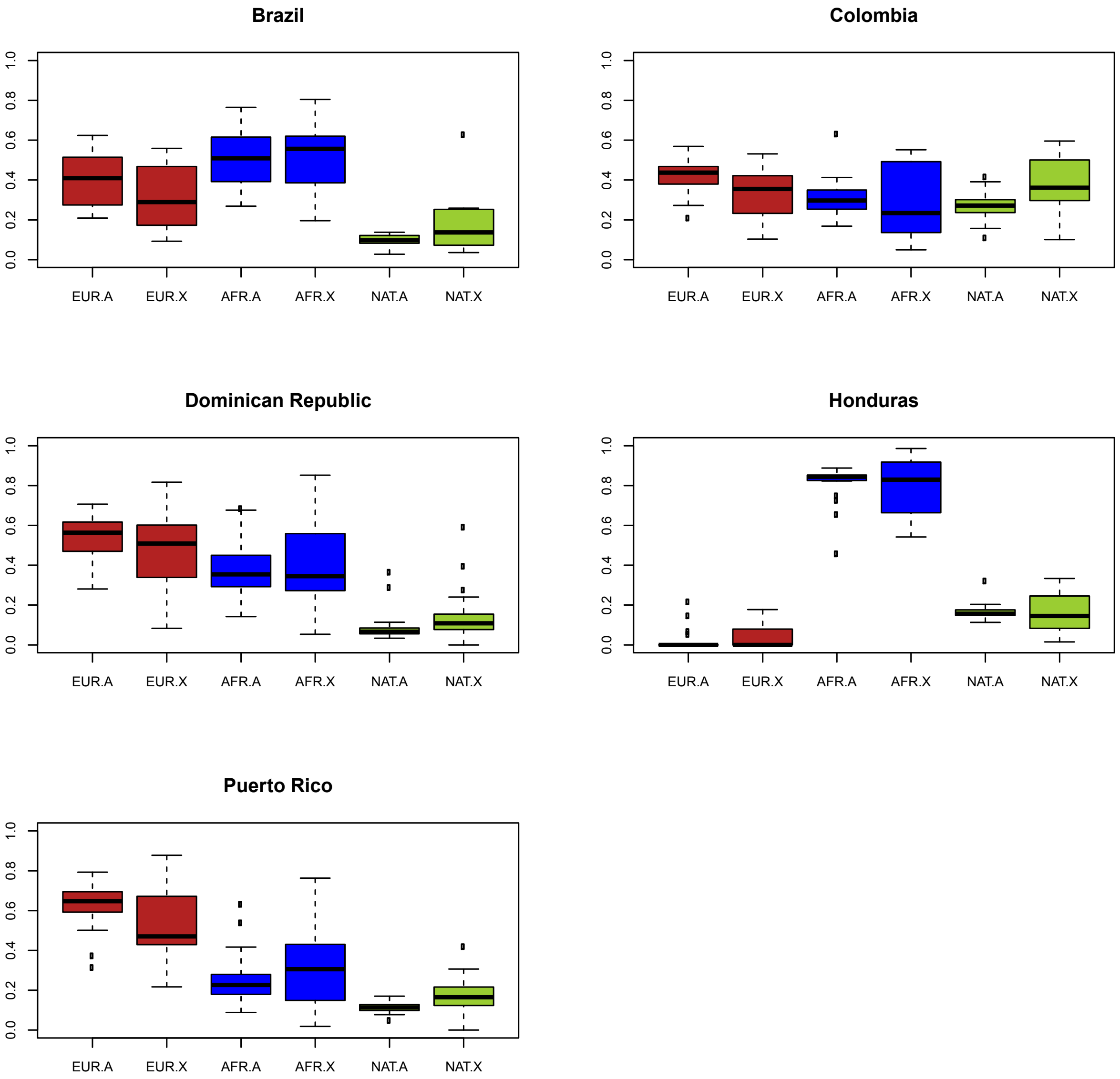

Supplementary Fig 12: Comparison of coverage on chromosome 22 for **[A]** All the variants in the whole genome; **[B]** Variants in exonic regions; **[C]** Variants in DNase I hypersensitive sites captured by commercial genotyping arrays. Bars represent the numbers of captured variants (**$r^2 \geq 0.5$**). From the left to the right, it shows 1) all variants identified by next generation sequencing technology (**Full**), 2) variants captured by other variants identified in sequencing data (i.e. if one variant fails to be sequenced, it can be inferred by other variants identified in sequencing data because of LD, **Sequence**), 3) variants captured by HumanOmni5-Quad BeadChip **(HumanOmni5M)**, 4) variants captured by **HumanOmni2.5M** BeadChip, 5) variants captured by **Human1M-Duo Beadchip**, 6) variants captured by Affymetrix Genome-Wide Human SNP Array 6.0 (**SNP Array 6.0)**, 7) variants captured by **Illumina HumanOmniExpress** BeadChip 8) variants captured by Illumina **Human Exome Beadchip**, and 9) variants captured by **Axiom**® **Exome** genotyping array, respectively. For each bar, the number of rare (0 < minor allele frequency [MAF] < 0.01), low-frequency (0.01 ≤ MAF < 0.05), and common (MAF ≥ 0.05) variants are shown from the dark to light colors. The proportions of captured rare, low-frequency and common variants are represented by red, green and blue lines, respectively.

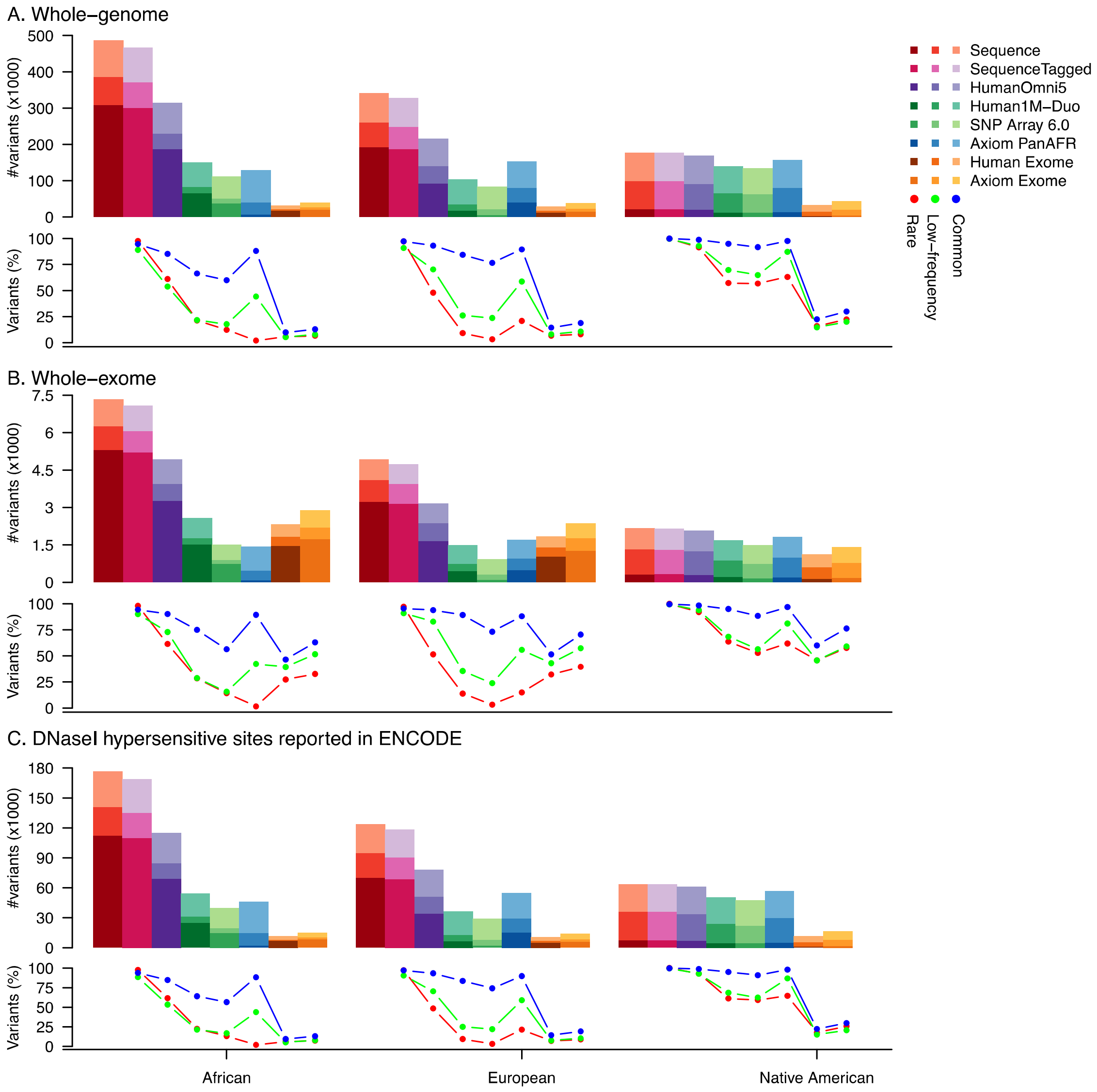

A. Whole−genome
B. Whole−exome
C. DNaseI hypersensitive sites reported in ENCODE
#variants (x1000)
Variants (%)
Sequence
SequenceTagged
HumanOmni5
Human1M−Duo
SNP Array 6.0
Axiom PanAFR
Human Exome
Axiom Exome
Rare
Low−frequency
Common
African
European
Native American

Supplementary Fig 13. Comparison of coverage on chromosome 22 for **[A]** All the variants in the whole genome; **[B]** Variants in exonic regions; **[C]** Variants in DNase I hypersensitive sites captured by commercial genotyping arrays. Bars represent the numbers of captured variants ($\boldsymbol{r^2 \geq 0.8}$). From the left to the right, this shows 1) all the variants identified by next generation sequencing technology (**Full**), 2) variants captured by other variants identified in sequencing data (i.e. if one variant fails to be sequenced, it can be inferred by other variants from sequencing data because of LD, **Sequence**), 3) variants captured by HumanOmni5-Quad BeadChip **(HumanOmni5M)**, 4) variants captured by **HumanOmni2.5M** BeadChip, 5) variants captured by **Human1M-Duo Beadchip**, 6) variants captured by Affymetrix Genome-Wide Human SNP Array 6.0 (**SNP Array 6.0)**, 7) variants captured by **Illumina HumanOmniExpress** BeadChip 8) variants captured by Illumina **Human Exome Beadchip**, and 9) variants captured by **Axiom®** **Exome** genotyping array, respectively. For each bar, the number of rare ($0 <$ minor allele frequency [MAF] < 0.01), low-frequency ($0.01 \leq$ MAF < 0.05), and common (MAF ≥ 0.05) variants are shown from the dark to light colors. The proportions of captured rare, low-frequency and common variants are represented by red, green and blue lines, respectively.

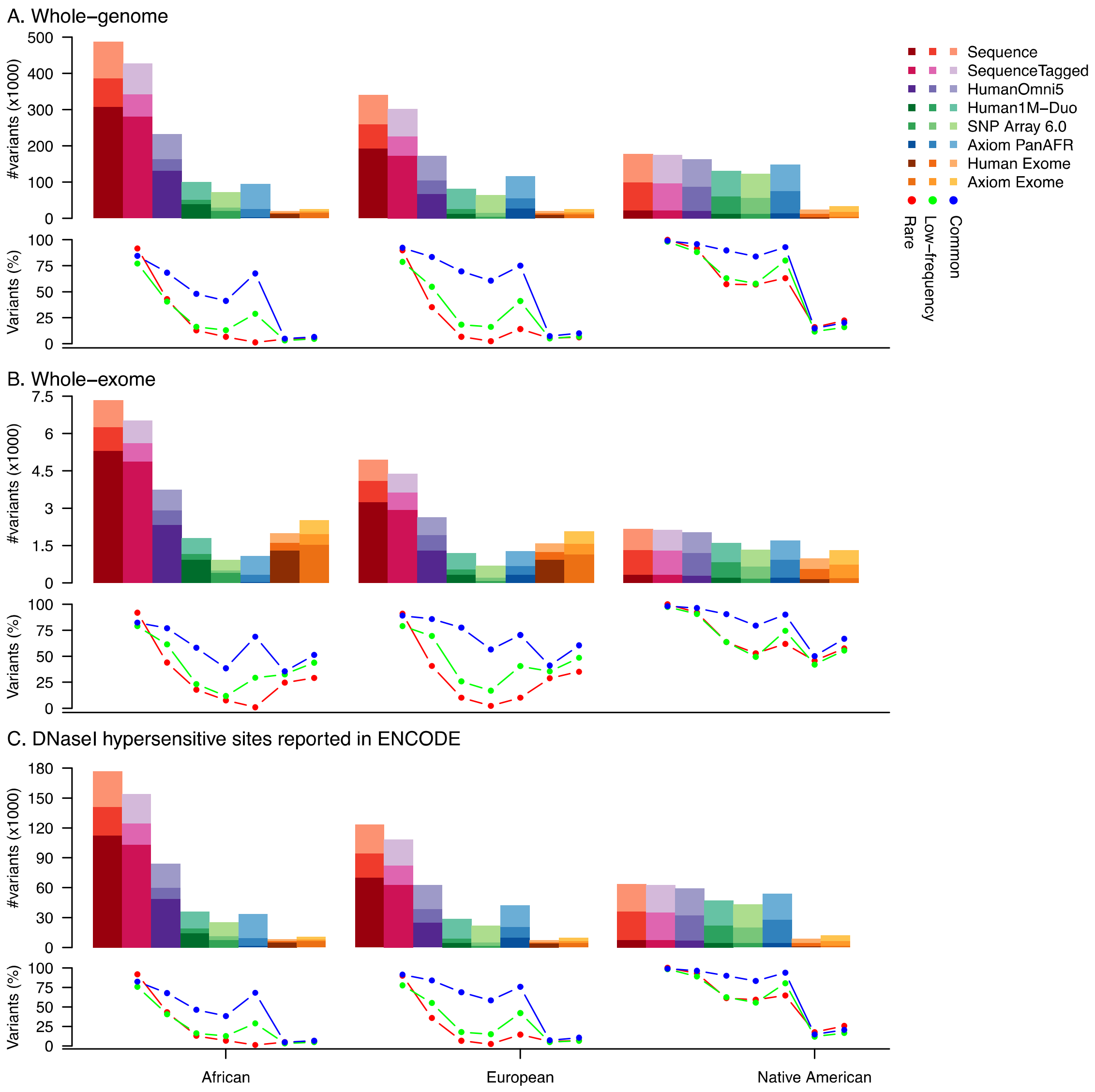

A. Whole−genome
#variants (x1000)
500
400
300
200
100
0
Variants (%)
100
75
50
25
0
B. Whole−exome
7.5
6
4.5
3
1.5
C. DNaseI hypersensitive sites reported in ENCODE
180
150
120
90
60
30
Sequence
SequenceTagged
HumanOmni5
Human1M−Duo
SNP Array 6.0
Axiom PanAFR
Human Exome
Axiom Exome
Rare
Low−frequency
Common
African
European
Native American

Supplementary Tables

Supplementary Table 1: Clinical characteristics and global estimates of ancestry for 642 individuals included in the final analysis set* for CAAPA by geographic sampling site and ethnicity. Global estimates of ancestry were obtained using protocols established by the 1000 Genomes Project[5] including the same set of 85 Utah residents with Northern and Western European ancestry (CEU), 88 Yoruba samples from Ibadan, Nigeria (YRI), and 43 Native Americans as reference populations. The Native Americans were selected from Mao et al.[2] with 99% or higher Native American ancestry and analysis was performed in ADMIXTURE[6] with K=3.

Ethnicity & Site	Total (% Male)	Asthma Status: Cases (% Male)	Controls (% Male)	Global estimates of ancestry from ADMIXTURE: African (YRI)	Case/ Control (YRI)	European (CEU)	Case/ Control (CEU)	Native American	Case/ Control (NatAM)
African American (Atlanta)	44 (63.6%)	22 (59.1%)	22 (68.2%)	84%	84%/85%	15%	15%/14%	1%	1%/1%
African American	50 (34.0%)	25 (44.0%)	25 (24.0%)	82%	81%/82%	17%	18%/16%	1%	1%/1%
African American	47 (53.2%)	24 (54.2%)	23 (52.2%)	76%	74%/79%	23%	25%/21%	1%	1%/1%^
African American	29 (55.2%)	15 (60.0%)	14 (50.0%)	82%	83%/80%	17%	16%/18%	2%	1%/3%
African American (NYC)	39 (56.4%)	18 (61.1%)	21 (52.4%)	81%	81%/81%	17%	18%/17%	2%	1%/2%
African American (Detroit)	26 (42.3%)	20 (40.0%)	6 (50.0%)	76%	76%/78%	22%	24%/19%	1%	1%/3%
African American (San	50 (60.0%)	25 (72.0%)	25 (48.0%)	76%	78%/74%	22%	21%/23%	2%	1%/3%
African American	43 (9.3%)	19 (5.3%)	24 (12.5%)	82%	84%/81%	17%	15%/18%	1%	1%/1%
Barbados	39 (56.4%)	22 (50.0%)	17 (64.7%)	84%	87%/81%	16%	13%/19%	0%	0%/0%
Jamaica	45 (51.1%)	23 (52.2%)	22 (50.0%)	89%	88%/90%	11%	11%/10%	1%	1%/0%
Dominican Republic (New	47(40.4%)	22(45.5%)	25 (36.0%)	38%	40%/37%	52%	52%/53%	9%	8%/10%
Honduras	41 (46.3%)	23 (56.5%)	18 (33.3%)	81%	80%/82%	2%	3%/1%	17%	17%/17%
Colombia	31 (51.6%)	13(61.5%)	18 (44.4%)	33%	35%/32%	39%	39%/39%	28%	26%/29%
Puerto Rico	53 (45.3%)	28 (60.7%)	25 (28.0%)	27%	31%/22%#	61%	58%/65%^	12%	12%/13%

Brazil	33 (45.5%)	6 (33.3%)	27 (48.2%)	49%	60%/46%^	41%	29%/44%^	10%	11%/10%
Nigeria	25 (48.0%)	15 (40.0%)	10 (60.0%)	99%	98%/100%	1%	2%/0%	0%	0%/0%
**All CAAPA**	**642 (46.9%)**	**20 (50.3%)**	**32 (43.5%)**	**70%**	**72%/68%**^	**24%**	**23%/26%**	**5%**	**5%/6%**^

* 18 individuals were dropped for relatedness and one sample was dropped due to absence of any African ancestry (African ancestry <0.1%).

# p < 0.01, two-sided t-test

^ p < 0.05, two-sided t-test

Supplementary Table 2: Summary of variation observed in 642 samples from CAAPA. Total variants, doubleton and singleton variant counts plus TiTv ratios generated at the SNV level across all bi-allelic SNVs in CAAPA; novel variation counts are generated at the allele level. Average counts of alleles by each category over all samples within each geographic sampling site / ethnicity. Average sequencing coverage and fraction of missed calls was first calculated within each individual and then averaged across individuals within a site, first based on data at all variable sites in the data set and then based on data only at the 551,510 autosomal SNPs used in the local admixture estimation.

						All variable sites		Sites used for admixture estimation	
**Ethnicity & Site**	**All Variants**	**Doubleton**	**Singleton**	**Novel alleles**	**TiTv Ratio**	**Sequence depth**	**Missing call rate**	**Sequence depth**	**Missing call rate**
African American (Atlanta)	3,937,257	16,855	32,744	83,893	2.15	34	7.6%	39	0.03%
African American (Chicago)	3,937,595	17,043	33,273	84,714	2.15	37	7.1%	43	0.07%
African American (Baltimore-DC)	3,902,693	17,036	33,417	81,390	2.15	35	7.2%	40	0.05%
African American (Nashville)	3,923,946	17,116	33,924	82,745	2.15	35	7.4%	40	0.03%
African American (NYC)	3,931,796	16,697	33,111	82,985	2.15	36	7.1%	42	0.06%
African American (Detroit)	3,882,718	17,036	33,727	79,741	2.15	33	7.6%	39	0.02%
African American (San Francisco)	3,907,208	17,039	34,517	81,413	2.15	36	7.2%	42	0.02%
African American (Winston-Salem)	3,916,211	16,964	32,700	82,853	2.15	36	7.4%	42	0.02%
Barbados	3,932,833	15,983	30,523	82,352	2.15	35	7.3%	41	0.04%
Jamaica	3,953,129	16,058	31,662	83,861	2.15	33	7.5%	38	0.07%
Dominican Republic (New York, Texas)	3,639,736	16,459	33,818	62,532	2.15	35	7.2%	41	0.05%
Honduras	3,903,654	13,913	15,946	80,805	2.15	36	7.3%	41	0.06%

Colombia	3,599,881	15,066	35,565	60,970	2.15	37	7.2%	43	0.24%
Puerto Rico	3,537,015	17,513	30,015	53,823	2.15	34	7.3%	40	0.03%
Brazil	3,728,654	17,850	29,302	67,906	2.15	35	7.1%	41	0.09%
Nigeria	3,971,632	13,620	26,545	78,920	2.15	32	7.8%	37	0.13%
**All CAAPA**	**3,850,372**	**16,390**	**31,299**	**76,931**	**2.15**	**35**	**7.3%**	**41**	**0.06%**

Supplementary Table 3: Summary of allele counts by annotation using SeattleSeq. Average counts of alleles by each category over all samples within each geographic sampling site / ethnicity.

	SeattleSeq Annotation						
**Ethnicity & Site**	**synonymous**	**missense**	**splice**	**Stop gained**	**Stop lost**	**3' UTR**	**5' UTR**
African American (Atlanta)	19,854	11,022	176	76	22	35,720	7,569
African American (Chicago)	19,818	10,998	175	74	22	35,592	7,566
African American (Baltimore-DC)	19,587	10,896	169	72	22	35,248	7,459
African American (Nashville)	19,734	10,995	171	75	22	35,544	7,530
African American (NYC)	19,792	11,024	172	73	22	35,542	7,556
African American (Detroit)	19,588	10,866	169	75	21	35,276	7,426
African American (San Francisco)	19,637	10,936	171	73	22	35,296	7,509
African American (Winston-Salem)	19,731	10,981	171	74	22	35,437	7,506
Barbados	19,834	11,034	175	74	22	35,692	7,579
Jamaica	19,998	11,115	172	74	22	35,965	7,653
Dominican Republic (New York, Texas)	18,362	10,286	163	76	20	32,894	6,983
Honduras	19,895	11,059	178	75	22	35,641	7,559
Colombia	18,264	10,195	165	69	20	32,658	6,953
Puerto Rico	17,942	10,066	160	73	20	32,222	6,792
Brazil	18,706	10,451	164	73	20	33,608	7,131
Nigeria	20,222	11,226	175	76	22	36,413	7,617
**All CAAPA**	**19,362**	**10,787**	**170**	**74**	**21**	**34,786**	**7,372**

Supplementary Table 4: Summary of SNVs private to each geographical sampling site within CAAPA. Comparison between geographic sampling sites is limited to SNVs that are non-monomorphic and bi-allelic in the full sample of 642 in CAAPA.

**Ethnicity & Site**	**All Variants**	**Singleton variants**	**MAF<0.01**	**0.01≤MAF≤0.05**	**MAF>0.05**
African American (Atlanta)	1,211,782	1,181,733	27,954	1,471	624
African American (Chicago)	1,404,826	1,365,656	34,998	2,912	1,260
African American (Baltimore-DC)	1,325,516	1,290,339	30,555	2,749	1,873
African American (Nashville)	826,918	812,711	12,855	975	377
African American (NYC)	1,081,319	1,059,375	19,090	1,908	946
African American (Detroit)	735,914	723,626	10,661	1,072	555
African American (San Francisco)	1,467,123	1,429,433	34,576	2,154	960
African American (Winston-Salem)	1,183,945	1,151,953	29,104	1,956	932
Barbados	987,381	959,402	25,532	1,666	781
Jamaica	1,190,861	1,159,336	29,031	1,702	792
Dominican Republic (New York, Texas)	1,387,414	1,311,602	73,511	1,613	688
Honduras	643,847	410,728	230,106	2,063	950
Colombia	954,025	917,153	32,896	2,135	1,841
Puerto Rico	1,456,993	1,278,276	176,365	1,646	706
Brazil	869,286	769,528	96,169	1,899	1,690
Nigeria	531,969	516,764	14,278	653	274

Supplementary Table 5: Variant annotation summary over all variants within CAAPA using ENCODE **[A]** and SeattleSeq **[B]**. **Annotation Key:** TSS = Transcription Start Site, PF = Promoter Flanking, E = Enhancer, WE = Weak Enhancer, CTCF = CTCF binding, T = Transcribed Region, R = Repressed or Inactive Region, notMod3 = number of coding bases is not a multiple of 3.

**[A] ENCODE Annotation**		
	**Count**	**%**
CTCF	435,138	1.01%
E	690,529	1.60%
None	2,346,332	5.43%
PF	22,360	0.05%
R	30,072,239	69.58%
T	9,305,252	21.53%
TSS	322,925	0.75%
WE	25,629	0.06%

**[B] SeattleSeq Annotation**		
	**Count**	**%**
coding-notMod3	607	0.00%
coding-synonymous	160,266	0.37%
coding-synonymous-near-splice	2,412	0.01%
intergenic	24,945,235	57.72%
intron	16,539,495	38.27%
missense	203,844	0.47%
missense-near-splice	3,390	0.01%
near-gene-3	447,961	1.04%
near-gene-5	482,913	1.12%
splice-3	828	0.00%
splice-5	1,182	0.00%
stop-gained	3,266	0.01%
stop-gained-near-splice	37	0.00%
stop-lost	195	0.00%
utr-3	344,661	0.80%
utr-5	84,097	0.19%
coding-notMod3-near-splice	15	0.00%

Supplementary Table 6: Summary of deleterious derived alleles determined using PhyloP (score at least 2.907), PolyPhen2 and ClinVar. PhyloP scores were generated excluding human reference, and variant sites where the reference allele was the derived allele were excluded from PolyPhen2 counts to accommodate biases arising at these sites in prediction algorithms. Average counts of alleles by each category over all samples within each geographic sampling site / ethnicity.

		Deleterious as defined by PhyloP scores								PolyPhen2 predictions				ClinVar count
		Counts				Fraction				Counts				
**Ethnicity & Site**	**% African**	**coding variants**	**non coding variants**	**UTR variants**	**All variants**	**coding variants**	**non coding variants**	**UTR variants**	**All variants**	**Benign**	**Possibly Damaging**	**Probably Damaging**	**Probably Damaging + SIFT < 0.05**	
African American (Atlanta)	84%	419	1615	83	2117	0.0135	0.0003	0.0018	0.0004	3643	983	1251	568	70
African American (Chicago)	82%	421	1612	86	2119	0.0136	0.0003	0.0018	0.0004	3620	981	1250	564	68
African American (Baltimore-DC)	76%	422	1614	83	2119	0.0136	0.0003	0.0018	0.0004	3595	976	1237	558	70
African American (Nashville)	82%	416	1616	83	2114	0.0134	0.0003	0.0018	0.0004	3627	989	1247	561	69
African American (NYC)	81%	419	1610	85	2114	0.0135	0.0003	0.0018	0.0004	3637	980	1254	569	70
African American (Detroit)	76%	415	1613	83	2111	0.0134	0.0003	0.0018	0.0004	3578	972	1236	558	70
African American (San Francisco)	76%	421	1609	84	2114	0.0135	0.0003	0.0018	0.0004	3604	975	1244	562	71
African American (Winston-Salem)	82%	415	1604	84	2103	0.0134	0.0003	0.0018	0.0004	3647	984	1236	556	68
Barbados	84%	420	1620	84	2124	0.0135	0.0003	0.0018	0.0004	3635	985	1259	568	70
Jamaica	89%	421	1613	87	2121	0.0136	0.0003	0.0019	0.0004	3675	984	1265	577	68
Dominican Republic (New York, Texas)	38%	429	1593	82	2104	0.0138	0.0003	0.0018	0.0004	3381	912	1180	522	72
Honduras	81%	418	1600	83	2101	0.0134	0.0003	0.0018	0.0004	3636	997	1261	576	69
Colombia	33%	429	1602	82	2113	0.0138	0.0003	0.0018	0.0004	3356	900	1170	519	68
Puerto Rico	27%	429	1606	81	2116	0.0138	0.0003	0.0017	0.0004	3308	892	1154	508	72

Brazil	49%	421	1607	81	2110	0.0136	0.0003	0.0017	0.0004	3444	927	1192	533	69
Nigeria	99%	416	1606	88	2110	0.0134	0.0003	0.0019	0.0004	3712	999	1269	578	68
**All CAAPA**	**70%**	421	1609	84	2113	0.0136	0.0003	0.0018	0.0004	3564	964	1231	554	70

Supplementary Table 7: Summary of deleterious derived allele counts determined using PhyloP (score at least 2.907) and PolyPhen2 by African/African European/European and Native American/Native American background. PhyloP scores were generated excluding human reference, and variant sites where the reference allele was the derived allele were excluded from PolyPhen2 counts to accommodate biases arising at these sites in prediction algorithms. Average counts of alleles by each category over all samples within each geographic sampling site / ethnicity.

Ethnicity & Site	PhyloP									PolyPhen2											
	African/ African			European/ European			Native American/ Native American			African/ African				European/ European				Native American/ Native American			
	coding	non coding	UTR	coding	non coding	UTR	coding	non coding	UTR	Probably Damaging	Possibly Damaging	Benign	Probably Damaging + SIFT < 0.05	Probably Damaging	Possibly Damaging	Benign	Probably Damaging + SIFT < 0.05	Probably Damaging	Possibly Damaging	Benign	Probably Damaging + [illegible]
African American (Atlanta)	302	1167	61	12	42	2	0	0	0	919	723	2688	340	29	21	80	10	0	0	0	0
African American (Chicago)	290	1107	60	15	57	3	0	0	0	875	692	2554	328	37	29	108	12	0	0	1	0
African American (Baltimore-DC)	247	965	50	26	96	5	0	0	0	761	604	2225	283	67	52	185	21	0	0	0	0
African American (Nashville)	277	1093	56	13	53	2	0	0	0	863	684	2526	321	36	30	107	10	0	0	0	0
African American (NYC)	286	1086	57	14	55	3	0	1	0	882	689	2541	328	39	29	115	12	0	0	2	0
African American (Detroit)	250	987	52	28	105	4	0	0	0	784	619	2262	293	76	60	217	24	0	0	1	0
African American (San Francisco)	251	969	50	25	88	5	0	1	0	775	609	2254	291	64	47	181	20	0	0	2	0
African American (Winston-Salem)	288	1127	61	19	74	4	0	0	0	875	703	2613	319	48	34	134	14	0	0	0	0
Barbados	305	1174	62	18	71	3	0	0	0	932	736	2704	345	47	35	132	14	0	0	0	0
Jamaica	335	1280	70	7	29	1	0	0	0	1009	794	2950	377	20	15	57	6	0	0	0	0
Dominican Republic (New York, Texas)	75	278	15	133	489	25	8	30	2	223	181	648	83	337	256	955	104	22	16	64	7
Honduras	270	1039	54	1	4	0	10	41	2	837	668	2432	306	3	2	9	1	25	21	77	7
Colombia	52	208	11	83	300	15	35	129	7	165	127	461	61	213	163	595	67	91	68	260	24

Puerto Rico	36	144	8	184	668	32	9	31	2	113	91	335	42	466	354	1321	149	24	18	64	6
Brazil	105	408	22	86	306	15	5	22	1	330	269	969	125	219	166	625	67	15	12	38	4
Nigeria	406	1569	86	1	3	0	0	0	0	1241	979	3629	457	2	1	5	0	0	0	0	0
**CAAPA**	**231**	**895**	**48**	**45**	**164**	**8**	**4**	**15**	**1**	**710**	**562**	**2072**	**264**	**114**	**87**	**324**	**36**	**10**	**8**	**30**	**3**

Supplementary Table 8: Difference in mean X-chromosomal and autosomal admixture fraction (%). For each population and for each ancestral group, the mean X-chromosomal admixture fraction minus the mean autosomal fraction (%) is listed. The Bonferroni-corrected paired t-test p-value follows in parenthesis. Positive values correspond to an excess of X-chromosomal ancestry and imply female-biased admixture; negative values correspond to an excess of autosomal ancestry and imply male-biased admixture. Values significant at the 0.05 level are in bold.

		Sample Size	YRI (*P*)	CEU (*P*)	Native American (*P*)
**All**		329	1.79 (5.52e-02)	-4.06 (8.94e-12)	2.27 (1.19e-12)
**African-American**		178	2.73 (3.21e-01)	-4.30 (5.64e-04)	1.57 (1.38e-05)
	Atlanta	16	1.50 (1.000)	-4.17 (1.000)	2.67 (1.000)
	Baltimore	22	8.17 (3.56e-01)	-9.43 (2.66e-02)	1.26 (1.000)
	Chicago	33	0.06 (1.000)	-1.39 (1.000)	1.33 (0.258)
	Detroit	17	-0.47 (1.000)	-2.01 (1.000)	2.48 (0.462)
	Nashville	14	8.04 (1.000)	-8.21 (1.000)	0.17 (1.000)
	NYC	17	3.49 (1.000)	-5.70 (1.000)	2.20 (1.000)
	San Francisco	20	2.43 (1.000)	-4.88 (1.000)	2.46 (1.000)
	North Carolina	39	1.73 (1.000)	-2.61 (1.000)	0.88 (1.000)
**Afro-Caribbean**		39	4.89 (1.000)	-6.33 (0.083)	1.43 (0.838)
	Barbados	17	7.28 (1.000)	-8.12 (1.000)	0.84 (1.000)
	Jamaica	22	3.05 (1.000)	-4.94 (1.000)	1.89 (1.000)
**Latin American**		112	1.36 (1.000)	-6.72 (5.83e-05)	5.36 (1.13e-05)
	Brazil	18	1.11 (1.000)	-8.96 (1.000)	7.85 (1.000)
	Colombia	15	-1.37 (1.000)	-9.49 (0.283)	10.86 (0.123)
	Dominican Republic	28	2.47 (1.000)	-7.50 (0.781)	5.03 (0.218)
	Honduras	22	-0.96 (1.000)	1.51 (1.000)	-0.55 (1.000)
	Puerto Rican	29	3.60 (1.000)	-9.38 (0.129)	5.78 (0.081)

Supplementary Table 9. Distinct mitochondrial haplotypes across CAAPA sites.

Population		African	European	Native American	Austro-nesian	East Asian	North African	South Asian	Canarian
African-American	Atlanta	41	3	0	0	0	0	0	0
	Baltimore	42	3	0	2	0	0	0	0
	Chicago	45	1	0	2	0	2	0	0
	Detroit	24	2	0	0	0	0	0	0
	Nashville	27	1	0	0	1	0	0	0
	NYC	36	2	1	0	0	0	0	0
	San Francisco	44	2	1	1	0	1	1	0
	North Carolina	38	4	0	1	0	0	0	0
Barbados		38	1	0	0	0	0	0	0
Brazil		18	4	11	0	0	0	0	0
Colombia		12	0	19	0	0	0	0	0
Dominican Republic		30	6	10	0	0	0	0	1
Honduras		31	1	9	0	0	0	0	0
Jamaica		45	0	0	0	0	0	0	0
Nigeria		24	1	0	0	0	0	0	0
Puerto Rican		17	5	31	0	0	0	0	0

Supplementary Table 10. Summary of Y-chromosomal haplotypes across all CAAPA sites (Supplementary Note 1.20). Ancestries corresponding to haplotypes listed in the first row are described in Supplementary Note 1.20. The most likely ancestry in the second row is denoted as Af=African, Eu=European, Na=Native American, Eu/As=European or Asian, Eu+Af=74 of these haplotypes are European and 2 are African.

Y-chromosomal haplotypes

Population	N	[redacted]												
All	**300**													
African American Atlanta	**28**		1		19			2	2		1	1		2
African American Baltimore	**25**			1	14			1	1					8
African American Chicago	**17**				13									4
African American Detroit	**9**			1	4		1		1					2
African American NYC	**22**	1	1		15	1		1						3
African American Nashville	**15**				12				1					2
African American San Francisco	**30**		1	1	16		1	1	1	2			2	5
African American North Carolina	**4**				1				1					2
Barbados	**22**			1	18	1					1			1
Brazil	**15**		1		3				4		1			6
Colombia	**16**				2		2	1	1					10
Dominican Republic	**19**				7						3			9
Honduras	**19**				15							3		1
Jamaica	**23**			1	14	1		1						6
Nigeria	**12**				12									
Puerto Rico	**24**				3		1	2	1	2				15

Supplementary Notes

Supplementary Note 1: Study populations

The study sample included N=642 individuals who passed all quality control criteria (see Notes 1.6 and 1.8, below) from a total of N=661 sequenced from 16 case-control studies of asthma participating in the NHLBI-supported *Consortium on Asthma among African-ancestry Populations in the Americas* (CAAPA; Table 1). The primary selection criteria applied to each study was (i) self-reported African ancestry; (ii) a physician's diagnosis of asthma or a negative history of asthma symptoms and asthma medication usage among controls; and (iii) the availability of at least 3 μg of DNA at a concentration at least 30 ng/μl from a primary blood sample. An overview of the final samples analyzed from each collection site is included below and in Supplementary **Table 1**.

Genomic Research on Asthma in the African Diaspora (GRAAD) study. GRAAD is a consortium comprised of 1,374 pediatric and adult African Americans with and without asthma, including one study on healthy African Americans, recruited through Johns Hopkins University and/or Howard University, in the Baltimore-Washington, D.C. metropolitan area[7-11]. Because asthma is characterized by onset during childhood, there was a deliberate decision to favor adults in the control group to minimize including controls with some potential for developing asthma; consequently, the mean age of GRAAD asthmatics is 23.78 (± 17.85) years and 35.23 (± 16.51) years for non-asthmatic controls. GRAAD subjects in the current study included 47 unrelated participants with unequivocal, current asthma (N=24) and non-asthmatic controls (N=23).

Reducing Emergency Asthma Care in Harlem (REACH) study. A population-based study of adult residents of Central or West Harlem who had visited the Harlem Hospital Center

Emergency Department (ED) for asthma exacerbation and who spoke English. During the REACH study recruitment period, 1,391 adults visited the ED for asthma and 726 patients (52%) were eligible. Non-asthmatic controls were selected from the same ED. Individuals from Harlem, New York City, with the full range of mild to moderate to severe asthma, were frequency-matched on age and gender with non-asthmatic controls[12-15]. REACH subjects in the current study included 39 unrelated participants with asthma (N=18) and non-asthmatic controls (N=21).

Study of African Americans, Asthma, Genes & Environments (SAGE II). SAGE II is an ongoing population-based, case-control study recruiting African American participants from clinics in the San Francisco Bay Area[16]. Subjects were eligible if they were 8-21 years of age, self-identified all four grandparents as African Americans, and had <10 pack-years of smoking history. Asthma was defined by physician diagnosis and report of symptoms in the 2 years preceding enrollment. Controls had no reported history of asthma or allergies during their lifetime. A total of 933 asthma cases and 646 controls have been recruited. SAGE II subjects in the current study included 50 unrelated participants with asthma (n=25) and non-asthmatic controls (n=25).

Bronchopulmonary Responses during Episodes of Asthma and the Treatment and History of Exacerbations (BREATHE) study. The BREATHE study is an NIAID-funded prospective observational study of 120 individuals ≥18 years old who were hospitalized for an acute asthma exacerbation at Vanderbilt University Medical Center from December 1999 to December 2003[17]. The subjects were initially identified by a presumptive admission diagnosis of an asthma exacerbation, and underlying asthma defined by physician diagnosis. Subjects were excluded if they had a life expectancy of <6 months, congestive heart failure or other active

chronic pulmonary disease, or previous enrollment in the study. BREATHE subjects in the current study included 15 randomly selected asthmatics with available DNA.

Validating biomarkers for Acute Lung Injury Diagnosis (VALID) study. Patients were selected from VALID, an observational cohort study of critically ill patients at risk for acute respiratory distress syndrome (ARDS) at the Vanderbilt Medical Center for the purpose of establishing a non-asthmatic control population representing the Nashville site. VALID has recruited critically ill patients since 2006, with a planned targeted enrollment of 2,550 patients . Inclusion criteria was admission to the Intensive Care Unit (ICU) with continued admission on day 2, and age greater than or equal to 18 years. Patients with chronic lung disease or uncomplicated overdose are excluded from VALID. VALID subjects in the current study included 14 critically ill African American adults with no prior medical history of asthma at the time of admission.

Chicago Asthma Genetics (CAG) study. CAG is a study of European American and African American families ascertained through affected sib pairs, case-parent trios (through affected offspring), adults and children with severe persistent asthma, and non-asthmatic control subjects (> 18 years)[14]. Asthma cases and families were recruited in the adult and/or pediatric asthma clinics at University of Chicago Hospital; controls were recruited from the medical center at large. CAG subjects in the current study included 50 unrelated African American participants with asthma (N=25) and non-asthmatic controls (N=25).

Study of Asthma Phenotypes and Pharmacogenomics Interactions by Race-ethnicity (SAPPHIRE). SAPPHIRE is a longitudinal, population-based study to identify the genetic predictors of asthma medication in a multi-ethnic patient (clinicaltrials.gov identifier, NCT01142947)[18]. Eligible patients received their care from a large, healthcare system serving

southeast Michigan and the greater Detroit metropolitan statistical area. Patients with asthma were ≥12 years of age, had a documented physician diagnosis of asthma in the medical record, and had no prior diagnosis of congestive heart failure or chronic obstructive pulmonary disease[19,20]. Control patients had the same enrollment criteria as case patients with the exception of having no prior diagnosis of asthma. The current study comprises a sample of SAPPHIRE participants with (N=25) and without (N=6) a diagnosis of asthma.

Severe Asthma Research Program (SARP). SARP was an NHLBI-supported study of non-smoking subjects with mild-to-severe asthma recruited at multiple centers in the U.S.[21,22]. Across the SARP network (and previous NHLBI Collaborative Study on the Genetics of Asthma at the Wake Forest site), 480 African American asthmatics and 289 African American healthy controls were studied. For the current study, 44 unrelated participants from the Wake Forest University (Winston-Salem, NC) site included adult asthmatics (N=19) and non-asthmatic controls (N=25), and 22 pediatric asthmatic participants were selected from the Emory University (Atlanta, Georgia) site.

Genetic Epidemiology of COPD (COPDGene) study. COPDGene is a 21-site, NHLBI-supported, multicenter study of the epidemiology and genetic determinants of chronic obstructive pulmonary disease (COPD) in European Americans and African Americans. Approximately 10,000 non-Hispanic whites and non-Hispanic African Americans with a history ≥ 10 pack-years of smoking, current or past, with and without COPD, without significant other concurrent lung disorders, and between the ages of 45 – 80 years have been enrolled[23-28]. Because non-asthmatic controls were not available for the Atlanta site, 22 non-asthmatic COPDGene subjects were included in the current study.

Barbados Asthma Genetics study (BAGS). A family-based and population-based study initiated in 1993 and comprised of 1,384 subjects (aged 30.63 ±17.06 years)[29],[30-39],[40-44]. Asthma probands were recruited through referrals at local polyclinics or the Accident and Emergency Department at the Queen Elizabeth Hospital in Bridgetown, Barbados, and nuclear and extended family members were subsequently recruited. To date 202 unrelated asthmatic and non-asthmatic controls have been recruited. BAGS subjects in the current study included 39 unrelated participants with unequivocal, current asthma (N=22) and non-asthmatic controls (N=17).

Jamaican Adolescent Asthma Study (JAAS). JAAS is a cross-sectional study on the prevalence of asthma and allergies in 897 Jamaican adolescents[45]. Participants' mothers were initially recruited into the Jamaica Perinatal Mortality Survey of 1986, which included all children born in Jamaica in September—October 1986. Children from Kingston, St. Andrew, and St. Catherine were contacted at ages 11 and 16 as part of a child development study, and again at age 18. JAAS subjects in the current study included 45 unrelated participants with unequivocal asthma (N=23) and non-asthmatic controls (N=22).

Genes-environments & Admixture in Latino Asthmatics (GALA II). GALA II is an ongoing multicenter case-control study of asthma in Latinos, organized from the coordinating center based at the University of California, San Francisco[16,46,47]. Cases and healthy controls of Latino ancestry were recruited from 5 urban study centers throughout the U.S. (Chicago, Illinois; Bronx, New York; Houston, Texas; San Francisco Bay Area, California; and San Juan, Puerto Rico). Subjects were recruited using the same protocols and phenotyping as described above for SAGE II, but in this case participants self-identified all four grandparents as Latinos. A total of 4,557 participants have been recruited (2,283 cases and 2,274 controls). Puerto Ricans GALA II

subjects in the current study included 53 individuals (28 with asthma and 25 without asthma) recruited in Puerto Rico who had both biological parents and all biological grandparents self-identified as Puerto Rican ethnicity. Dominican GALA II participants in this study included 47 individuals (22 with asthma and 25 without asthma) recruited in New York (n=46) and Texas (n=1), and they reported having both biological parents and all biological grandparents self-identified as Dominicans, except for two individuals that had one of their parents being from a different Latino subgroup.

The Honduras Genetics of Asthma in Non-European Populations (HONDAS) study. HONDAS is a population-based study of asthma and population dynamics, structure, and phylogenetic relations of the Garífuna (Black Carib) people from the northern coast of Honduras, a population of African and Red Carib Native Amerindian ancestry[48,49]; and of Honduran autochthonous Amerindian populations of Mesoamerican or South American ancestry. To date, 858 subjects have been recruited from 12 villages (Bajamar, Travesía, Corozal, Sambo Creek, Alfonzo Lacayo, Belén Gualcho, San Juan, Tornabé, Triunfo de la Cruz, Cristales, Río Negro, and Santa Fe ; aged 5-85). HONDAS subjects in the current study include 41 unrelated Garífuna participants with asthma (N=23) and non-asthmatic controls (N=18).

Proyecto Genes Candidatos en Asma (PGCA) study. PGCA is a population-based study conducted by the Institute for Immunological Research of The University of Cartagena (Colombia) to identify environmental and genetic risk factors for asthma and allergies[50-54]. A total of 836 unrelated asthma cases and 574 non-asthmatic controls were recruited from the Social Security Clinic and outpatient health centers in Cartagena from 2002 to 2005. An additional 655 individuals in 167 nuclear and extended families were recruited through an

asthmatic proband. PGCA subjects in the current study included 31 unrelated participants with asthma (N=13) and non-asthmatic controls (N=18).

Brazilian Immunogenetics of Asthma and Schistosomiasis (BIAS) study. BIAS a whole-population ascertainment designed study of asthma and schistosomiasis in the rural district of Conde, Bahia, located 200 km north of Salvador, Brazil[53,55-58]. Subjects were recruited July – September, 2004, from five communities (Buri, Camarao, Genipapo, Sempre Viva, and Cobo), and follow-up visits continue. To date, 822 subjects have been enrolled from an estimated population of 1,700. The dataset is comprised of 2 large pedigrees with 535 and 310 members collapsed into 318 nuclear families (aged 5-85 years). BIAS subjects in the current study included 33 unrelated participants with asthma (N=6) and non-asthmatic controls (N=27).

Social Changes, Asthma, and Allergies in Latin America (SCAALA) study. SCAALA is a longitudinal study involving a cohort of 1,445 children living in Salvador, Brazil, enrolled in a city-wide sanitation program when they were 0-3 years old (1996-2003) [53,59-62] Data on asthma and allergic diseases and potential risk factors associated with a poor, urban, tropical environment have been collected in successive surveys[63]. The prevalence of asthma is 22.6% in this cohort (N=327 asthma cases)[59]. A total of 48 SCAALA participants were originally included in the sequenced samples for CAAPA, but due to data sharing issues were dropped from the results presented in this paper. Quality control metrics described in Supplementary Notes 1.11 and 1.12 as well as Supplementary Fig 2 and Supplementary Fig 3 include these 48 samples; however all analysis pertaining to variant counts, annotation, global and local ancestry de-convolution, deleterious burden load, etc. have been performed excluding these subjects.

Asthma, Environment and the Genes Study (AEGS). AEGS is a population-based study of genetic variation and environmental factors contributing to risk and severity of asthma

in Yoruba-speaking children aged 6 to 18 years living in rural and urban settings in Southwest Nigeria[64,65]. In Phase I, a total of 1,690 school children living in rural communities were recruited, including 104 asthmatics. An additional 121 non-asthmatic 'control' subjects were enrolled and matched with asthma cases based on sex and age (1:1 ratio). AEGS subjects in the current study included 25 unrelated participants with asthma (N=15) and non-asthmatic controls (N=10).

Supplementary Note 2: Informed consent

All study participants in the whole genome sequencing study provided written informed consent for the use of their DNA in genetic studies. A careful review was conducted to verify that the consents were consistent with the activities of this study. Institutional review board approval was obtained at Johns Hopkins University (GRAAD, BAGS, BIAS, HONDAS, PGCA), Howard University (GRAAD), Columbia University (REACH), Wake Forest University (SARP), Morehouse School of Medicine (COPDGene), Henry Ford Health System (SAPPHIRE), the University of California, San Francisco (coordinator center for SAGE II and GALA II), the Western Institutional Review Board for the recruitment in Puerto Rico (GALA II Puerto Ricans), Baylor College of Medicine from Texas, Albert Einstein College of Medicine Yeshiva University, Jacobi Medical Center, the North Central Bronx Hospital from New York (GALA II Dominicans), Children's Hospital and Research Center Oakland and Kaiser Permanente-Vallejo Medical Center (SAGE II), Vanderbilt University (BREATHE, VALID), the University of Chicago (CAG, AEGS), University of the West Indies, Mona campus (JAAS) and Cave Hill Campus, Barbados (BAGS), The University of Cartagena (PGCA), the Universidad Católica de Honduras in San Pedro Sula (HONDAS), the Federal University of Bahia and endorsed by the

National Commission for Ethics in Human Research in Brazil (BIAS, SCAALA), and The University of Ibadan, Nigeria (AEGS).

Supplementary Note 3: DNA quality and whole genome sequencing

Genomic DNA was quantified prior to library construction using PicoGreen (Quant-iT™ PicoGreen® dsDNA Reagent, Invitrogen). DNA quantities were read with Spectromax Gemini XPS (Molecular Devices). PCR-Free paired-end libraries were manually generated from 500ng–1ug of gDNA using the Illumina TruSeq DNA Sample Preparation Kit according to the TruSeq DNA PCR-Free Sample Preparation Guide. Pre-fragmentation gDNA cleanup was performed using paramagnetic sample purification beads (Agencourt® AMPure® XP reagents, Beckman Coulter). Samples were fragmented and libraries were size selected following fragmentation and end-repair using paramagnetic sample purification beads, targeting 300 bp inserts. Final libraries were quality controlled for size using a gel electrophoretic separation system and are quantified. Following library quantitation, DNA libraries were denatured, diluted, and clustered onto v3 flow cells using the Illumina cBot™ system. cBot runs were performed based on the cBot User Guide, using the reagents provided in Illumina TruSeq Cluster Kit v3. Clustered v3 flow cells were loaded onto HiSeq 2000 instruments and sequenced (30x coverage) on 100 bp paired-end, non-indexed runs. All samples were sequenced on independent lanes. Sequencing runs were performed based on the HiSeq 2000 User Guide, using Illumina TruSeq SBS v3 Reagents. Illumina HiSeq Control Software (HCS) and Real-Time Analysis (RTA) used on HiSeq 2000 sequencing runs for real-time image analysis and base calling.

Supplementary Note 4: Variant calling and generation of a multi-sample vcf

Assembly of each individual genome was performed using the Consensus Assessment of Sequence and Variation (CASAVA) package[66]. During the build process, CASAVA collates, filters, and compiles aligned reads. CASAVA then calls the genomic consensus sequence using a probabilistic algorithm and compares it to the reference sequence to identify homozygous or heterozygous SNPs. The SNP-caller implemented in the Illumina CASSAVA module employs a probabilistic model which ultimately produces probability distributions over all diploid genotypes for each site in the genome. The primary values summarized from these distributions are a set quality scores: Q(SNP) and Q(max_gt). The value of Q(SNP) expresses the probability that any SNP exists at the site, *i.e.* the probability that the genotype at this site is not the homozygous reference state. The value of Q(max_gt) expresses the probability of the most-likely genotype state at this site, reported as the value 'max_gt'. Note the value Q(max_gt) corresponds to a value referred to as 'consensus quality' in SNP-calling methods such as 'samtools pileup'.

The values for both Q(SNP) and Q(max_gt) are designed for any application which requires a general survey of all sites in the genome. One additional score is provided by the SNP-caller which can be used to look at sites where there is a strong expectation that the site is polymorphic. This value is Q(max_gt|poly_site), which expresses the probability of the most-likely genotype state at the site, assuming the site is polymorphic. This state is separately reported as the value 'max_gt|poly_site'. This genotype value and quality score provides greater sensitivity when looking at, for example, a particular set of polymorphic sites from dbSNP. This value should not be used to evaluate the genotype for every position in the genome as this would result in a high number of false positive SNP predictions, and therefore was not utilized in the analysis of the CAAPA data.

To accommodate diverse applications, the CASAVA variant caller does not filter out low-confidence calls and thus prints all sites where Q(SNP) is greater than zero to the " SNPs.txt" file. For calls with a very low Q(SNP) score, it is possible the most likely genotype will be the homozygous reference, *e.g*. max_gt will be 'CC' for a position with a reference value of 'C'. This can be interpreted to mean there is a non-trivial probability of a heterozygous SNP existing at this site, but that the homozygous reference genotype is still more likely than any non-reference variant. In CAAPA all sites with Q(SNP)<20 were filtered.

SNP-caller methods summary: The SNP-caller calculates probability distributions over all diploid genotype states in several steps. These steps include several forms of noise filtration, read re-alignment, heuristic adjustment of same-strand base call quality to reflect potential error dependencies between calls, and finally calculation of genotype probabilities via a Bayesian model. The procedure is outlined below.

As a first step the variant caller filters out reads from both SNP and indel-calling based on a number of criteria. First, any reads marked as failing primary analysis quality checks (*e.g* failing the purity filter) or marked as a PCR or optical duplicates are removed from consideration. Next, for paired-end reads any reads not marked as being part of a 'proper pair' are removed from consideration. This is intended to remove any reads from chimeric pairs, with unmapped mates or with an anomalous pair insert size. Next, reads are filtered on alignment mapping quality. For paired-end reads the SNP-caller removes by default any read with a paired-end mapping quality less than 90, and for single-end reads, those with a single-end mapping quality less than 10 are removed.

After this initial read filtering, the variant caller proceeds with candidate indel discovery and generation of alternate read alignments based on these candidate indels (this is described in

more detail in the indel caller Supplementary Note below). As part of this re-alignment process the variant-caller selects a representative alignment to be used for site genotype calling and depth summarization by the SNPcaller. This alignment is selected to be within a certain threshold of the most-likely of all alignments for a read, and any leading or trailing portions of the read with ambiguous support for 2 or more different alignments are marked as clipped. This representative alignment does not affect the indel caller – the indel calling process considers all alignments for each read without end-clipping.

Given the set of filtered and realigned reads, the variant caller next runs filtering on base calls within these reads. First, any contiguous trailing sequence of 'N' base calls are effectively treated as trimmed off of the ends of reads for the purpose of genotyping and depth calculation. Next, the mismatch density filter is run on all reads to mask out sections of the read having an unexpectedly high number of disagreements with the reference. The current default mismatch density filter behavior is as follows:

• Base calls are ignored where more than 2 mismatches to the reference sequence occur within 20 bases of the call. Note, this filter treats each insertion or deletion as a single mismatch.

• If the call occurs within the first or last 20 bases of a read, then the mismatch limit is applied to the 41 base window at the corresponding end of the read.

• The mismatch limit is applied to the entire read when the read length is 41 or shorter.

All bases marked by the mismatch density filter, together with any 'N' base calls which remain after the end-trimming step, are filtered out by the variant caller. These filtered base calls are not used for site genotyping but appear in the filtered base call counts in the variant caller's output for each site.

All remaining base calls are used for site-genotyping. The genotyping method heuristically adjusts the joint error probability calculated from multiple observations of the same allele on each strand of the genome to account for the possibility of error dependencies between these observations. The method accomplishes this by treating the highest quality base call of each allele from each strand as independent observations, leaving their associated base call quality scores unmodified. However, subsequent base calls for each allele and strand have their qualities adjusted to increase the joint error probability of that allele above the error expected from independent base call observations.

After running the site-genotyper on all positions, a set of candidate SNP sites is produced, consisting of all sites with Q(SNP) > 0. A final filtration step is taken to remove potentially spurious SNP-calls which occur near the centromeres and other high-copy number regions. This is done by calculating the mean used depth for each chromosome, and filtering out all SNP calls which occur at a used depth which is greater than 3 times this chromosomal mean.

Data processing to generate a multi-sample VCF file for each chromosome from the Illumina MAXGT single-sample SNP VCF files provided in Illumina's standard deliverable package (sample/Variations/SNPs.vcf.gz) was performed at Knome, Inc. (Cambridge, MA, USA). The individual VCF files were merged using VCFtools v0.1.11[67]. Using custom scripts, the multi-sample VCF files were backfilled to include homozygous reference genotypes and depth of coverage from the sites.txt files (sample/Assembly/Parsed/chr/chr.sites.txt.gz). Custom QC scripts confirmed that the multi-sample VCFs and the single-sample VCFs had the same number of heterozygous and homozygous alternate genotypes. VCFtools [1] was used to confirm that all subjects were included in each multi-sample VCF. The multi-sample VCF was generated including the 48 samples from SCAALA, but these were subsequently dropped from all analysis

(aside from QC metric presented in Supplementary Notes 7 and 8 as well as Supplementary Fig 2 and Supplementary Fig 3).

Supplementary Note 5: Robustness of results using single- versus multi-sample calling algorithms

To compare the results of the single-sample variant calling used with a multi-sample calling method, we applied the Real-Time Genomics multi-sample caller[68] to our full set of samples on chromosome 22. Quality assessment of a subset of the two sets of calls based on a Random Forests classifier trained using the available Illumina Omni genotyping array data revealed the single-sample calls provided equal or better call quality than the multi-sample calls. In addition, replication of the analysis of deleterious allele counts by subject based on the multi-sample calls revealed identical patterns to those shown in this manuscript using the single-sample calls. Based on these analyses, we are confident in the call quality and the conclusions drawn in this manuscript based on our single-sample variant calls.

Supplementary Note 6: Identity-by-descent (IBD) analysis

We estimated the amount of DNA shared identical by descent (IBD) using PLINK software on all 661 sequenced individuals from CAAPA. IBD analyses were performed prior to the generation of the multi-sample VCF by KNOME, Inc. (Supplementary Note 1.4, above) and were performed relying on genotype data generated as part of the Illumina Omni 2.5 genotype array performed on all 709 samples at Illumina. To conduct these analyses, we used a subset of 490,179 linkage disequilibrium (LD) pruned markers using a window size of 50, step of 5 and r^2 threshold of 0.3. The following classifications were made where Z0=P(IBD=0), Z1=P(IBD=1),

and Z2=P(IBD=2): (1) subject pairs were classified as duplicates if Z2>0.97; (2) subject pairs were classified as parent-offspring if Z1>0.97, and Z0 and Z2 < 0.05; (3) subject pairs were classified as full siblings if Z0<0.4, Z1>0.4, and Z2>0.16; (4) subject pairs were classified as half siblings if Z0>0.6, Z1<0.58 and Z2<0.05; (5) subject pairs were classified as first cousins if Z0>0.6, Z1<0.4, and Z2<0.02; (6) subject pairs were classified as unrelated if Z0>0.78. As illustrated in **Supplementary Fig 1,** based on these definitions 18 samples were dropped from further analysis: (1) one duplicate pair was identified in Honduras and both individuals were removed from further analyses as we were unable to resolve the perfect match of DNA to sample ID in the absence of any additional genotype data; (2) 6 parent-offspring pairs (4 from Conde, Brazil and 2 from Barbados) were identified, and one member from each pair was removed from further analyses; (3) two full sibling pairs were identified from Honduras and one member from each pair was removed from analyses; (4) 8 half-sibling pairs were identified (4 from Conde, Brazil, 2 from Honduras, and 2 from Baltimore) and one member from each pair was dropped; (5) there was one pair with high relatedness (π_hat=0.5012) from Honduras that did not match any of the above classifications and one subject from this pair was also removed.

Supplementary Note 7: Quality control with respect to genotype quality and depth filtering

To select genotype quality (Q) and depth (DP) cutoffs, we performed a concordance analysis using genotypes from the Illumina Omni 2.5 arrays provided by Illumina for each of our sequenced samples. We considered all autosomal loci on the array where we could resolve the alleles on the array with those from the sequencing data, and where we received calls from the sequencing data set, meaning the position had at least one non-reference allele in at least one individual. We calculated concordance values, focusing on calls that were either heterozygous or

homozygous non-reference on the Omni array, and calculated the fraction of Omni genotypes correctly called (detection rate) and the fraction of incorrect sequencing calls (1 - accuracy). We then considered two possible Q cutoffs, 20 and 30, and two possible DP cutoffs, 7 and 10, based on recommendations by Illumina and our collaborators. As these filters are applied, there is a tradeoff between decreasing the detection rate (since fewer calls will be made) and increasing the accuracy. Ideally, a filter would provide a large increase in accuracy for a relatively small decrease in detection.

Supplementary Fig 2 shows results comparing no filter with Q20 and Q30 filters (top row), DP7 and DP10 filters (middle row) and a combination of Q20 with either DP7 or DP10 (bottom row). Detection rate is shown on the y-axis and 1-accuracy is shown on the x-axis. Comparing Q20 with no filter, we see a relatively modest loss of detection relative to the improvement in accuracy, particularly for heterozygous calls. The additional filtering of variants with quality between 20 and 30 does not show as much gain in accuracy, and a relatively greater loss of detection. We see similar results comparing no filter to DP7 and DP10 filters, and when we combined the Q20 filter with the DP7 and DP10 filters. Given these results, we filtered variants with $Q < 20$ and $DP < 7$, since these filters provided some improvement in accuracy for a relatively modest decrease in detection rate.

Supplementary Note 8: Quality control with respect to segmental duplication region filtering

Regions of segmental duplication can be enriched for genotype call errors due to the challenges of aligning short reads to duplicated parts of the genome. In our data set, we observed a pattern of excess heterozygote calls in segmental duplications (**Supplementary Fig 3**, top row) as

illustrated by increased counts of heterozygous samples per variant position. We also observed inflation in the count of variable sites in regions of segmental duplication (**Supplementary Fig 3**, bottom row, left panel). By removing variants falling into these regions of segmental duplication, we reduced this inflation and observed a relatively even spread of variant density across entire length of chromosome 22 (**Supplementary Fig 3**, bottom row, right panel). For equality in comparison of variant sites between the 1000 Genomes Project and CAAPA, we applied the same segmental duplication filter to the 1000 Genomes Project data.

Supplementary Note 9: Quality control with respect to individual sequencing depth and variant call missingness

After performing the above filtering steps, for each individual in the data set, we calculated the average sequencing depth at all sites polymorphic in CAAPA, and the fraction of these sites with missing genotype calls. We performed the same analysis with the subset of data restricted to the 551,510 sites used in the local ancestry estimation (see Methods for details). Results are shown in **Supplementary Table 2**, as averaged across individuals in a sampling site / ethnicity. Sensitivity analysis was performed for the main results presented by adjusting for individual depth and missingness and no difference was observed in terms of the patterns or significance of the results.

Supplementary Note 10: SNP annotation

SNP annotation was performed using the SeattleSeq Annotation server[69]; SNPs were annotated as coding-notMod3, coding-synonymous, coding-synonymous-near-splice, intergenic, intron,

missense, missense-near-splice, near-gene-3, near-gene-5, splice-3, splice-5, stop-gained, stop-gained-near-splice, stop-loss, utr-3, utr-5, and coding-notMod3-near-splice.

We also annotated the CAAPA variation using functional segmentation calls of ENCODE data. ChromHMM and Segway, two unsupervised learning algorithms[70,71], were used to make functional predictions from 13 experiments (including CHiP-Seq, DNase-seq, and FAIRE-seq) on multiple cell lines and were combined giving seven predicted categories: Predicted promoter region including transcription start site (TSS), Predicted promoter flanking region (PF), Predicted enhancer (E), Predicted weak enhancer or open chromatin *cis* regulatory element (WE), Transcriptional repressor_CTCF enriched element (CTCF,), Predicted transcribed region (T), and Predicted Repressed or Low Activity region (R). We downloaded these data from http://ftp.ebi.ac.uk/pub/databases/ensembl/encode/integration_data_jan2011/byDataType/segmentations/jan2011/hub/ on March 18, 2013. Consensus calls were made between non-cancerous cell lines.

Supplementary Note 11: Estimations of coverage for commercial arrays

To compare the coverage performances of commercial arrays, we considered 565,244 bi-allelic SNVs in chromosome 22 with call rates 80% or higher and that were under Hardy-Weinberg equilibrium (HWE; $P \geq 10^{-9}$). Linkage disequilibrium (LD) was measured by pair-wise r^2 for sites with African-, European-, and Native American-ancestry, respectively. We defined a SNV as captured if the maximum r^2 between this SNV and any other "tagging" SNVs within a 500kb window was more than or equal to a given threshold (e.g. $r^2 \geq 0.5$ or $r^2 \geq 0.8$). The tagging SNVs could be other variants in sequencing data, variants in genome-wide genotyping arrays (e.g. Illumina HumanOmni5-Quad BeadChip, Illumina HumanOmni2.5 BeadChip, Illumina

Human1M-Duo Beadchip, Affymetrix Genome-Wide Human SNP Array 6.0 and Illumina HumanOmniExpress BeadChip), and variants in genotyping arrays focusing on exonic variants (e.g. Illumina Human Exome BeadChip and Axiom® Exome genotyping array). Comparisons were conducted based on variants identified in the whole-genome, on variants in exonic regions, and on variants in regulatory regions (*e.g.* DNase I hypersensitive sites summarized in ENCODE).

For each site, we have inferred its local ancestry, which may come from a single population (i.e., with African-, European-, and Native American-ancestry) or from two different populations. If the site comes from the same population, we assign the genotype of this site to this population with a weight of 1.0. However, if the site comes from two different populations, we assign the genotype of this site to both populations but with a weight of 0.5 for each population. For the calculation of pair-wise r^2, we only consider genetic variants located within a physical distance of 250kb of each other. We estimate r^2 by maximizing the log-likelihood function,

$$\ln L \sim (2N_{AABB}+N_{AABb}+N_{AaBB})\ln p_{AB}+(2N_{AAbb}+N_{AABb}+N_{Aabb})\ln p_{Ab}+(2N_{aaBB}+N_{AaBB}+N_{aaBb})\ln p_{aB}+(2N_{aabb}+N_{Aabb}+N_{aaBb})\ln p_{ab}$$
$$+N_{AaBb}\ln(p_{AB}p_{ab}+p_{Ab}p_{aB})$$

where N_{G1G2} is the observed weighted number for different combinations of genotypes of two sites; p_{AB}, p_{Ab}, p_{aB}, and p_{ab} are the expected haplotype frequencies which need to be estimated based on the maximum log-likelihood. The pair-wise r^2 can be calculated as $r^2 = \frac{p_{AB}-p_A p_B}{p_A p_a p_B p_b}$, where p_A, p_a, p_B, and p_b is the allele frequency.

In the CAAPA sequencing data, more variants were captured in the African-ancestral chromosomal regions compared with those in European- and Native American-ancestral regions (Supplementary **Figs 12 and 13**). This was expected given the higher diversity in African-

ancestral populations compared to other populations, because the effective population size for the African-ancestral components in this study was highest among these three groups and all non-African populations experienced some bottlenecks associated with the serial Out-of-Africa migrations. Not surprisingly, because of higher LD in Native American and European ancestry populations, a larger proportion of variants can be tagged by genotyping arrays in non-African components compared to African-ancestral chromosomal components. Focusing on the African-ancestral component, we observed 94.7% of common variants (MAF $\geq$ 0.05), 89.1% of low-frequency variants (0.01 $\leq$ MAF < 0.05) and 97.6% of rare variants (0 < MAF < 0.01) could be captured by other variants in WGS data under the cutoff of $r^2 \geq 0.5$. Similarly, the capture fraction for the European-ancestral component is 97.3% for common variants, 90.8% for low-frequency variants, and 97.2% for rare variants. Nearly all variants (i.e., 99.8% for common, 99.6% for low-frequency and 99.98% for rare ones) can be captured for the Native American-ancestral component, which is consistent with their most recent migration and spread into the New World.

For the African-ancestral components, although as high as 85.2% of common variants could be captured by tag SNPs in standard GWAS arrays (e.g. HumanOmni5), less than 62% of low-frequency and rare variants could be captured using these arrays (Supplementary **Fig 12A**). Not surprisingly, because of higher LD in Native American and European ancestry populations, a larger proportion of variants can be tagged by genotyping arrays in non-African components compared to African-ancestral chromosomal components. For example, less than 94%, 71% and 48% of common, low frequency and rare variants can be captured by the GWAS arrays in the European-ancestral components, and less than 99%, 93% and 92% of common, low frequency

and rare variants (respectively) can be captured in Native American ancestral component (Supplementary **Fig 12A**).

This comparison between variants tagged by variants from WGS and GWAS arrays illustrates how a higher sensitivity can be achieved in capturing low frequency and rare variants by WGS data. This pattern is also seen at a stricter cutoff of $r^2 \geq 0.8$. (Supplementary **Fig 13**).

We further explored the HumanExome Beadchip and Axiom® Exome genotyping arrays, which focus only on exonic variants, and found more exonic variants could be captured by these arrays compared to common GWAS arrays. For example, only HumanOmni5 was comparable with exome genotyping arrays in the capture fraction of exonic variants, while ~20 times more tag SNPs were used in HumanOmni5. As observed in the GWAS arrays, exonic genotyping arrays underperformed in capturing the low frequency and rare variants, compared with common variants, while the performance of sequencing data is consistent for all types of variants (rare, low-frequency and common; Supplementary **Figs 12B and 13B**). We also evaluated the performance of genotyping arrays on the capture of regulatory variants (such as variants in DNaseI hypersensitive sites), compared with WGS data. Using the CAAPA sequencing data, we found most variants in DNaseI hypersensitive sites, especially low frequency and rare variants, are not captured by any of the current genotyping arrays (Supplementary **Figs 12C and 13C**). While not unexpected, this observation underscores the value of the CAAPA catalog as an important and unique reference database in designing genotyping arrays customized for African ancestral populations, given the importance of regulatory elements and their contribution to human disease and evolution.

Supplementary References

1 Patterson, N., Price, A. L. & Reich, D. Population structure and eigenanalysis. *PLoS Genet* **2**, e190 (2006).

2 Mao, X. *et al.* A genomewide admixture mapping panel for Hispanic/Latino populations. *Am J Hum Genet* **80**, 1171-1178 (2007).

3 Bigham, A. W. *et al.* Andean and Tibetan patterns of adaptation to high altitude. *Am J Hum Biol* **25**, 190-197 (2013).

4 1000 Genomes Project Consortium. A map of human genome variation from population-scale sequencing. *Nature* **467**, 1061-1073 (2010).

5 Consortium, G. P. *FTP directory /vol1/ftp/technical/working/20130711_native_american_admix_train/ at ftp.1000genomes.ebi.ac.uk*, <ftp://ftp.1000genomes.ebi.ac.uk/vol1/ftp/technical/working/20130711_native_american_admix_train/> (2014).

6 Alexander, D. H., Novembre, J. & Lange, K. Fast model-based estimation of ancestry in unrelated individuals. *Genome Res* **19**, 1655-1664 (2009).

7 Hizawa, N. *et al.* Linkage analysis of *Dermatophagoides pteronyssinus* (Der p)-specific IgE responsiveness with polymorphic markers on chromosome 6p21 (HLA-D region) in Caucasian families by Transimssion/Disequilibrium Test (TDT). *Journal of Allergy and Clinical Immunology* **102(3)**, 342-448 (1998).

8 Hizawa, N. *et al.* Genetic regulation of *Dermatophagoides pteronyssinus* (Der p)-specific IgE responsiveness: A genome-wide multipoint linkage analysis in families recruited through two asthmatic sibs. *Journal of Allergy and Clinical Immunology* **102(3)**, 436-442 (1998).

9 Hizawa, N. *et al.* Genetic influences of chromosomes 5q31-q33 and 11q13 on specific IgE responsiveness to common inhaled allergens among African American families. *Journal of Allergy and Clinical Immunology* **102(3)**, 449-453 (1998).

10 Blumenthal, M. N. *et al.* A genome-wide search for allergic response (atopy) genes in three ethnic groups: Collaborative Study on the Genetics of Asthma. *Human Genetics* **114(2)**, 157-164 (2004).

11 Blumenthal, M. N. *et al.* Genome scan for loci linked to mite sensitivity: the Collaborative Study on the Genetics of Asthma (CSGA). *Genes and Immunity* **5(3)**, 226-231 (2004).

12 Ford, J. G. *et al.* Patterns and predictors of asthma related emergency department use. *Chest* **120**, 1129-1135 (2001).

13 Pesola, G. R. *et al.* Predicting asthma morbidity in Harlem emergency department patients. *Academic Emergency Medicine* **11**, 944-950 (2004).

14 Torgerson, D. G. *et al.* Genome-wide ancestry association testing identifies a common European variant on 6q14.1 as a risk factor for asthma in African American subjects. *J Allergy Clin Immunol* **130**, 622-629 e629 (2012).

15 Flores, C. *et al.* African ancestry is associated with asthma risk in African Americans. *PLoS ONE* **7**, e26807 (2012).

16 Nishimura, K. K. *et al.* Early-life air pollution and asthma risk in minority children. The GALA II and SAGE II studies. *Am J Respir Crit Care Med* **188**, 309-318 (2013).

17 Arnold, D. H., Gebretsadik, T., Minton, P. A., Higgins, S. & Hartert, T. V. Clinical measures associated with FEV1 in persons with asthma requiring hospital admission. *Am J Emerg Med* **25**, 425-429 (2007).

18 Padhukasahasram, B. *et al.* Gene-based association identifies SPATA13-AS1 as a pharmacogenomic predictor of inhaled short-acting beta-agonist response in multiple population groups. *Pharmacogenomics J* (2014).

19 Gould, W. *et al.* Factors predicting inhaled corticosteroid responsiveness in African American patients with asthma. *J Allergy Clin Immunol* **126**, 1131-1138, doi:10.1016/j.jaci.2010.08.002 (2010).
20 Rumpel, J. A. *et al.* Genetic ancestry and its association with asthma exacerbations among African American subjects with asthma. *J Allergy Clin Immunol* **130**, 1302-1306, doi:10.1016/j.jaci.2012.09.001 (2012).
21 Moore, W. C. *et al.* Characterization of the severe asthma phenotype by the National Heart, Lung, and Blood Institute's Severe Asthma Research Program. *J Allergy Clin Immunol* **119**, 405-413 (2007).
22 Jarjour, N. N. *et al.* Severe asthma: lessons learned from the National Heart, Lung, and Blood Institute Severe Asthma Research Program. *Am J Respir Crit Care Med* **185**, 356-362, doi:10.1164/rccm.201107-1317PP (2012).
23 Regan, E. A. *et al.* Genetic epidemiology of COPD (COPDGene) study design. *COPD* **7**, 32-43, doi:10.3109/15412550903499522 (2010).
24 Washko, G. R. *et al.* Lung volumes and emphysema in smokers with interstitial lung abnormalities. *N Engl J Med* **364**, 897-906, doi:10.1056/NEJMoa1007285 (2011).
25 Foreman, M. G. *et al.* Early-onset chronic obstructive pulmonary disease is associated with female sex, maternal factors, and African American race in the COPDGene Study. *Am J Respir Crit Care Med* **184**, 414-420, doi:10.1164/rccm.201011-1928OC (2011).
26 Castaldi, P. J. *et al.* The association of genome-wide significant spirometric loci with chronic obstructive pulmonary disease susceptibility. *Am J Respir Cell Mol Biol* **45**, 1147-1153, doi:10.1165/rcmb.2011-0055OC (2011).
27 Kim, D. K. *et al.* Genome-wide association analysis of blood biomarkers in chronic obstructive pulmonary disease. *Am J Respir Crit Care Med* **186**, 1238-1247, doi:10.1164/rccm.201206-1013OC (2012).
28 Hansel, N. N. *et al.* Racial differences in CT phenotypes in COPD. *COPD* **10**, 20-27, doi:10.3109/15412555.2012.727921 (2013).
29 Barnes, K. C. *et al.* Linkage of asthma and total serum IgE concentration to markers on chromosome 12q: Evidence from Afro-Caribbean and Caucasian populations. *Genomics* **37**, 41-50 (1996).
30 Barnes, K. *et al.* Linkage analysis of atopy to chromosome 12q markers in an Afro-Caribbean population. *Journal of Allergy and Clinical Immunology* **99**, S476 (1997).
31 Barnes, K. C. *et al.* Dense mapping of chromosome 12q13.12-q23.3 and linkage to asthma and atopy. *Journal of Allergy and Clinical Immunology* **104(2)**, 485-491 (1999).
32 Lonjou, C. *et al.* A first trial of retrospective collaboration for positional cloning in complex inheritance: Assay of the cytokine region on chromosome 5 by the Consortium on Asthma Genetics (COAG). *Proceedings of the National Academy of Sciences* **97(20)**, 10942-10947 (2000).
33 Nickel, R. *et al.* Atopic dermatitis is associated with a functional mutation in the promoter of the CC chemokine RANTES. *Journal of Immunology* **164**, 1612-1616 (2000).
34 Barnes, K. C. *et al.* Testing for gene-gene interaction controlling total IgE in families from Barbados: evidence of sensitivity regarding linkage heterogeneity among families. *Genomics* **71(2)**, 246-251 (2001).
35 Liang, K. Y., Hsu, F. C., Beaty, T. H. & Barnes, K. C. Multipoint linkage-disequilibrium-mapping approach based on the case-parent trio design. *American Journal of Human Genetics* **68(4)**, 937-950 (2001).
36 Palmer, L. *et al.* A retrospective collaboration on chromosome 5 by the International Consortium on Asthma Genetics (COAG). *Clin Exp Allergy* **31**, 152-154 (2001).

37 Hsu, F. C., Liang, K. Y., Beaty, T. H. & Barnes, K. C. Unified sampling approach for multipoint linkage disequilibrium mapping of qualitative and quantitative traits. *Genetic Epidemiology* **22**, 298-312 (2002).
38 Manolio, T. A. *et al.* Sex differences in heritability of sensitization to Blomia tropicalis in asthma using regression of offspring on midparent (ROMP) methods. *Human Genetics* **113(5)**, 437-446 (2003).
39 Manolio, T. A. *et al.* Correlates of sensitization to BLOMIA TROPICALIS and DERMATOPHAGOIDES PTERONYSSINUS in asthma in Barbados. *International Archives of Allergy and Immunology* **131(2)**, 119-126 (2003).
40 Pillai, S. G. *et al.* A genome-wide search for linkage to asthma phenotypes in the genetics of asthma international network families: evidence for a major susceptibility locus on chromosome 2p. *Eur J Hum Genet* (2006).
41 Webb, B. T. *et al.* Quantitative linkage genome scan for atopy in a large collection of Caucasian families. *Hum Genet* **121**, 83-92 (2007).
42 Pillai, S. G. *et al.* Factor analysis in the Genetics of Asthma International Network family study identifies five major quantitative asthma phenotypes. *Clin Exp Allergy* **38**, 421-429 (2008).
43 White, J. H. *et al.* Identification of a novel asthma susceptibility gene on chromosome 1qter and its functional evaluation. *Hum Mol Genet* **17**, 1890-1903 (2008).
44 Zambelli-Weiner, A. *et al.* Evaluation of the CD14/-260 polymorphism and house dust endotoxin exposure in the Barbados Asthma Genetics Study. *J Allergy Clin Immunol* **115**, 1203-1209 (2005).
45 Knight-Madden, J., Forrester, T. E., Hambleton, I. R., Lewis, N. & Greenough, A. Skin test reactivity to aeroallergens in Jamaicans: relationship to asthma. *West Indian Med J* **55**, 142-147 (2006).
46 Borrell, L. N. *et al.* Childhood obesity and asthma control in the GALA II and SAGE II studies. *Am J Respir Crit Care Med* **187**, 697-702 (2013).
47 Kumar, R. *et al.* Factors associated with degree of atopy in Latino children in a nationwide pediatric sample: the Genes-environments and Admixture in Latino Asthmatics (GALA II) study. *J Allergy Clin Immunol* **132**, 896-905 e891 (2013).
48 Herrera-Paz, E. F., Garcia, L. F., Aragon-Nieto, I. & Paredes, M. Allele frequencies distributions for 13 autosomal STR loci in 3 Black Carib (Garifuna) populations of the Honduran Caribbean coasts. *Forensic Sci Int Genet* **3**, e5-10 (2008).
49 Herrera-Paz, E. F., Matamoros, M. & Carracedo, A. The Garifuna (Black Carib) people of the Atlantic coasts of Honduras: Population dynamics, structure, and phylogenetic relations inferred from genetic data, migration matrices, and isonymy. *Am J Hum Biol* **22**, 36-44 (2010).
50 Caraballo, L., Cadavid, A. & Mendoza, J. Prevalence of asthma in a tropical city of Colombia. *Annals of Allergy* **68**, 525-529 (1992).
51 Vergara, C. & Caraballo, L. Asthma mortality in Columbia. *Ann Allergy Asthma Immunol* **80**, 55-60 (1998).
52 Vergara, C. *et al.* Association of G-protein-coupled receptor 154 with asthma and total IgE in a population of the Caribbean coast of Colombia. *Clin Exp Allergy* **39**, 1558-1568 (2009).
53 Vergara, C. *et al.* African Ancestry Is a Risk Factor for Asthma and High Total IgE Levels in African Admixed Populations. *Genetic Epidemiology* **37**, 393-401 (2013).
54 Vergara C, T. Y., Grant AV, Rafaels N, Gao L, Hand T, Stockton M, Campbell M, Mercado D, Faruque M, Dunston G, Beaty TH, Oliveira RR, Ponte EV, Cruz AA, Carvalho E, Araujo MI, Watson H, Schleimer RP, Caraballo L, Nickel RG, Mathias RA, Barnes KC. Gene encoding Duffy antigen/receptor for chemokines is associated with asthma and IgE in three populations. *American Journal of Respiratory and Critical Care Medicine* **178**, 1017-1022 (2008).

55 Grant, A. V. *et al.* High heritability but uncertain mode of inheritance for total serum IgE level and Schistosoma mansoni infection intensity in a schistosomiasis-endemic Brazilian population. *J Infect Dis* **198**, 1227-1236 (2008).
56 Grant, A. V. *et al.* Polymorphisms in IL10 are associated with total IgE levels and *Schistosoma mansoni* infection intensity in a Brazilian population. *Genes and Immunity* **in press** (2010).
57 Oliveira, R. R. *et al.* Factors associated with resistance to Schistosoma mansoni infection in an endemic area of Bahia, Brazil. *Am J Trop Med Hyg* **86**, 296-305 (2012).
58 Grant, A. V. *et al.* Functional polymorphisms in IL13 are protective against high Schistosoma mansoni infection intensity in a Brazilian population. *PLoS ONE* **7**, e35863, doi:10.1371/journal.pone.0035863 PONE-D-11-20730 [pii] (2012).
59 Cunha, S. S. *et al.* Asthma cases in childhood attributed to atopy in tropical area in Brazil. *Rev Panam Salud Publica* **28**, 405-411 (2010).
60 Kakkar, R. & Lee, R. T. The IL-33/ST2 pathway: therapeutic target and novel biomarker. *Nat Rev Drug Discov* **7**, 827-840 (2008).
61 Dinarello, C. A. The biological properties of interleukin-1. *Eur Cytokine Netw* **5**, 517-531 (1994).
62 Dinarello, C. A. Interleukin-18, a proinflammatory cytokine. *Eur Cytokine Netw* **11**, 483-486 (2000).
63 Barreto, M. L. *et al.* Risk factors and immunological pathways for asthma and other allergic diseases in children: background and methodology of a longitudinal study in a large urban center in Northeastern Brazil (Salvador-SCAALA study). *BMC Pulm Med* **6**, 15, doi:10.1186/1471-2466-6-15 (2006).
64 Ige, O. M. *et al.* Atopy is associated with asthma in adults living in rural and urban southwestern Nigeria. *J Asthma* **48**, 894-900, doi:10.3109/02770903.2011.608458 (2011).
65 Oluwole, O. *et al.* Allergy sensitization and asthma among 13-14 year old school children in Nigeria. *African Health Sciences* **13(1)**, 144-153 (2013).
66 CASAVA. (Illumina Inc, 2014).
67 Danecek, P. *et al.* The variant call format and VCFtools. *Bioinformatics* **27**, 2156-2158 (2011).
68 Cleary, J. G. *et al.* Joint variant and de novo mutation identification on pedigrees from high-throughput sequencing data. *Journal of computational biology : a journal of computational molecular cell biology* **21**, 405-419, doi:10.1089/cmb.2014.0029 (2014).
69 SeattleSeq. *SeattleSeq Annotation 129*, <http://snp.gs.washington.edu/SeattleSeqAnnotation129/> (2014).
70 Hoffman, M. M. *et al.* Unsupervised pattern discovery in human chromatin structure through genomic segmentation. *Nat Methods* **9**, 473-476 (2012).
71 Ernst, J. & Kellis, M. Discovery and characterization of chromatin states for systematic annotation of the human genome. *Nat Biotechnol* **28**, 817-825 (2010).
